# Mutators can drive the evolution of multi-resistance to antibiotics

**DOI:** 10.1371/journal.pgen.1010791

**Published:** 2023-06-13

**Authors:** Danna R. Gifford, Ernesto Berríos-Caro, Christine Joerres, Marc Suñé, Jessica H. Forsyth, Anish Bhattacharyya, Tobias Galla, Christopher G. Knight

**Affiliations:** 1 Division of Evolution, Infection and Genomics, School of Biological Sciences, Faculty of Biology, Medicine and Health, The University of Manchester, Manchester, United Kingdom; 2 Department of Earth and Environmental Sciences, School of Natural Sciences, Faculty of Science and Engineering, The University of Manchester, Manchester, United Kingdom; 3 Department of Physics and Astronomy, School of Natural Sciences, Faculty of Science and Engineering, The University of Manchester, Manchester, United Kingdom; 4 Department of Evolutionary Theory, Max Planck Institute for Evolutionary Biology, Plön, Germany; 5 Department of Evolutionary Ecology and Genetics, Christian-Albrechts-University of Kiel, Kiel, Germany; 6 Instituto de Física Interdisciplinar y Sistemas Complejos, IFISC (CSIC-UIB), Campus Universitat Illes Balears, Palma de Mallorca, Spain; Institut Cochin, FRANCE

## Abstract

Antibiotic combination therapies are an approach used to counter the evolution of resistance; their purported benefit is they can stop the successive emergence of independent resistance mutations in the same genome. Here, we show that bacterial populations with ‘mutators’, organisms with defects in DNA repair, readily evolve resistance to combination antibiotic treatment when there is a delay in reaching inhibitory concentrations of antibiotic—under conditions where purely wild-type populations cannot. In populations of *Escherichia coli* subjected to combination treatment, we detected a diverse array of acquired mutations, including multiple alleles in the canonical targets of resistance for the two drugs, as well as mutations in multi-drug efflux pumps and genes involved in DNA replication and repair. Unexpectedly, mutators not only allowed multi-resistance to evolve under combination treatment where it was favoured, but also under single-drug treatments. Using simulations, we show that the increase in mutation rate of the two canonical resistance targets is sufficient to permit multi-resistance evolution in both single-drug and combination treatments. Under both conditions, the mutator allele swept to fixation through hitch-hiking with single-drug resistance, enabling subsequent resistance mutations to emerge. Ultimately, our results suggest that mutators may hinder the utility of combination therapy when mutators are present. Additionally, by raising the rates of genetic mutation, selection for multi-resistance may have the unwanted side-effect of increasing the potential to evolve resistance to future antibiotic treatments.

## Author summary

The global rise in antimicrobial resistance means we urgently need new approaches that halt its spread. Combination therapy, using multiple antibiotics as one treatment, proposes to do just that. Evolving resistance to combinations should be exceedingly rare, as it requires multiple mutations to occur in the same genetic background before microbial growth is inhibited. We find that wild-type bacteria cannot achieve this, even when inhibition does not occur rapidly. However, we show that introducing a small number of ‘mutators’ defective in DNA repair allows multi-drug resistance to readily evolve during both single-drug and combination treatments. As mutators are common in natural populations and infections, our results suggest that combination therapy may not be as resilient against resistance evolution as once thought.

## Introduction

Rising rates of resistance and declines in antimicrobial discovery have lead to an emerging public health crisis. Consequently, there is an urgent need for strategies that suppress resistance evolution to preserve existing antimicrobials. There has been sustained interest in the use of ‘combination therapy’ to prevent the development of resistance in infectious diseases. [1–3] and cancer [4]. Combination therapy uses multiple drugs as part of the same treatment, an approach that has proved successful in various settings [5–9]. There is significant interest in expanding the use of combination therapy to tackle the global burden of antimicrobial resistance [10–12]. Considerable attention has been given to characterising how combinations inhibit bacterial growth [13–15], especially through exploiting non-additive effects (i.e. synergy and antagonism [3, 13, 16–20]) and higher-order interactions [21]. However, there is currently conflicting evidence that combining therapies can stop the emergence of resistance, with combinations performing no better than monotherapy in some contexts [22, 23]. Determining what governs the resilience of combinations against resistance evolution therefore remains an open question.

A fundamental principle of combination therapy is that a single lineage must acquire multiple independent resistance mechanisms (‘multi-resistance’) to achieve full resistance. Under ideal conditions, multi-resistance should be prevented by two factors. First, mutation rates to resistance are small enough [24] that spontaneously acquiring multi-resistance should be exceedingly rare [1, 25]. Second, inhibition by multiple drugs should prevent multi-resistance from being acquired sequentially, as single-resistant lineages are prevented from reproducing and acquiring subsequent resistances.

However, variation in microbial populations, and the environments they inhabit, means that these ideals may not always be achieved during antibiotic use. For instance, mutation rates can vary considerably within and between populations. In particular, ‘mutators’ with 10- to 1000-fold higher mutation rates are often present at frequencies as high as 30% in natural populations (e.g. infections and host-associated microbiomes [26–31]). Furthermore, variation in antibiotic concentrations over time and space can expose populations to sub-inhibitory environments (e.g. due to a time-lag between treatment and inhibition [32–34], variation in concentrations between tissues [35], or treatment non-adherence [36, 37]). This allows sensitive individuals to reproduce and generate resistant mutants, which subsequently increase in frequency due to positive selection (the ‘mutant selection window’ [38, 39]). Although combination therapy has been proposed as a solution to the problems imposed by the mutant selection window and by mutators [39], evidence for the efficacy of combinations against mutators is mixed [40–42], including an experimental study where mutators inoculated at a frequency of 100% were capable of evolving multi-resistance *in vivo* [43]. These studies raise several questions about how populations are able to overcome combination treatment, if it depends on initial mutator frequency, and what genomic mechanisms and evolutionary processes are involved. Understanding the potential for mutators to evolve multi-resistance during antibiotic treatment, and the evolutionary dynamics at play, is therefore crucial for predicting when and how combination therapies will be effective in preventing resistance.

Here, we investigate whether a sub-population of mutators can enable multi-resistance evolution under conditions involving a time-lag between the application of antibiotics and inhibition. Using a combination of experimental evolution and stochastic modelling, we assessed whether the presence of mutators could allow populations to evolve multi-resistance. We established populations of *Escherichia coli* with frequencies of mutators found in nature, and exposed them to environments with rifampicin and nalidixic acid, either alone or in combination. Early work on combinations suggested this particular combination should be resilient against resistance [44], and modern combinations also frequently involve drugs from the same antibiotic classes (i.e. rifamycins [45] and fluoroquinolones [46]). Hence, this combination is a useful model system for assessing the role of mutators in evolving resistance to combinations.

Both experimental and simulation results showed that the presence of mutators can significantly enhance the evolution of multi-resistance. Sequencing revealed that multi-resistant isolates from the combination treatment possessed mutations in the canonical targets of each drug [47, 48], as well as additional variation across the genome; in most isolates, this included changes to multi-drug efflux pumps, and in some, there were additional defects in DNA replication and repair systems. Simulations allowed us to test targeted hypotheses about the evolutionary dynamics of multi-resistance that would be difficult to test experimentally. Specifically, we demonstrated that the initial mutator allele is sufficient to enable multi-resistance to emerge through its effects on mutation rates of the canonical targets of each drug.

Remarkably, experimental and simulation results both showed the emergence of multi-resistance in response to single-drug as well as combination therapies. Our findings demonstrate that this is a result of the spread of the mutator allele along with single-drug resistance, leading to an elevated mutation rate that enables the emergence of multi-resistance even when it is not beneficial. For one of two single-drug treatments, the emergence of multi-resistance is equal to or greater than for the combination treatment. However, only the combination treatment ultimately facilitated the spread of resistance once it had emerged. Overall, our results imply that multi-resistance evolution in mutators may represent a significant obstacle to the widespread deployment of combination therapy against bacterial infection, given the prevalence of mutators in natural microbial populations.

## Results

### Multi-resistance evolves in both single-drug and combination treatments when mutators are present

To determine the conditions under which multi-resistance evolves, we performed experimental evolution using four mutator frequency treatments (none, low, intermediate, high) and four selection regimes (antibiotic-free, single-drug with either rifampicin or nalidixic acid, combination with both antibiotics). We employed ramping selection, where antibiotic concentrations were doubled daily over six days (from 0.625 mg/l to 20 mg/l of each drug, where 10 mg/l of either is sufficient to inhibit wild-type growth). This concentration range is physiologically relevant for these antibiotics [49, 50]. We conducted daily assays to detect resistance, which is defined as the ability for a random sample of the population to grow on selective media containing the antibiotic(s) at concentrations above the minimum inhibitory concentration (MIC) of the wild-type strain. This characterises five possible outcomes: no resistance (no growth on any selective media), single resistance (growth on only one of two single-drug selective media), mixed resistance (growth on both single-drug selective media, but not in combination media), and double resistance, i.e. multi-resistance to two antibiotics (growth in both single-drug and combination selective media).

**Fig 1:**
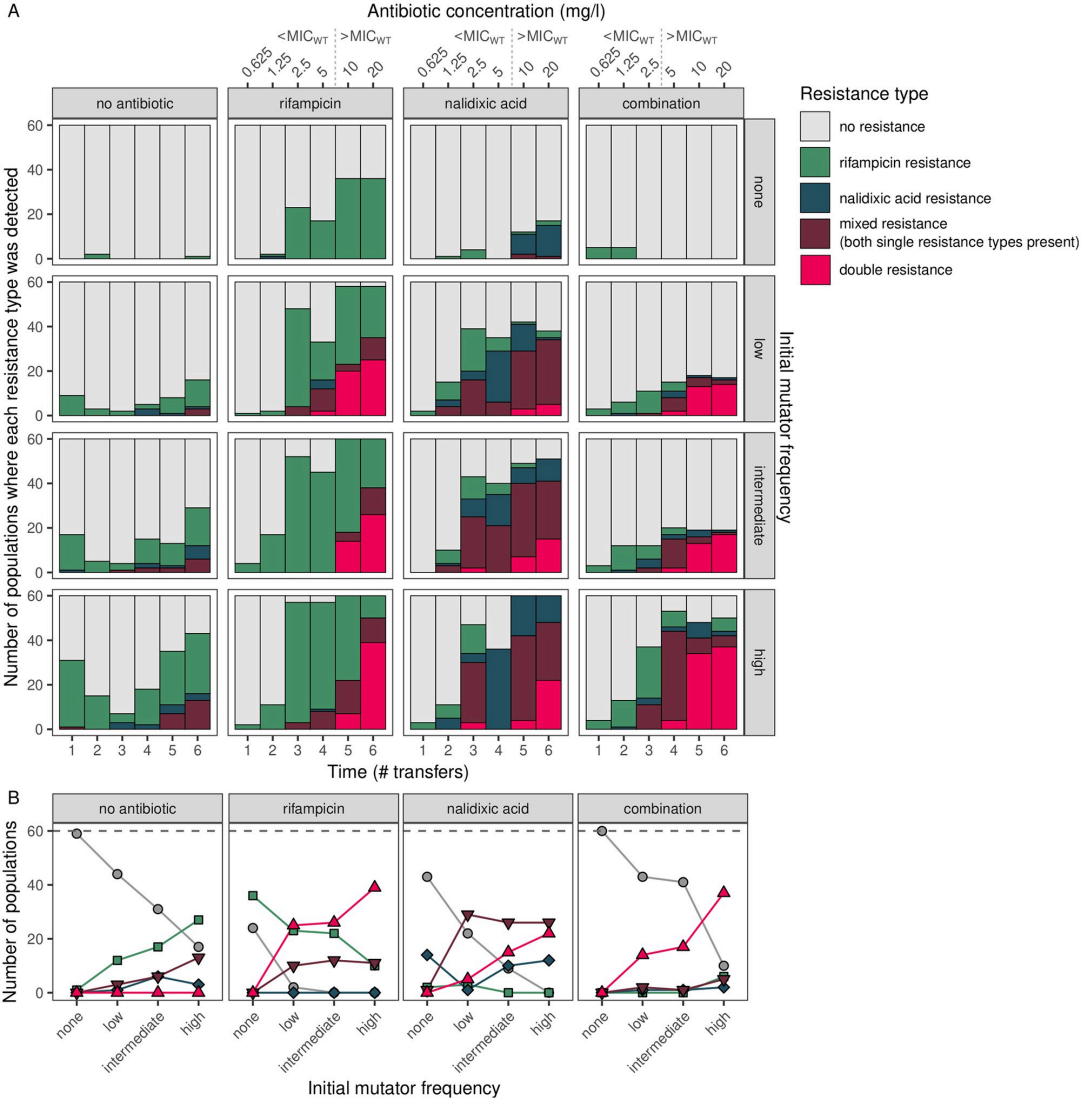
Multi-resistance evolves in populations where mutators are present in both single-drug and combination drug treatments. A. Dynamics over the course of the experiment. B. Summary of outcomes across treatments after the final transfer. ‘Resistance type’ is characterised by growth on single-drug (‘rifampicin resistance’, ‘nalidixic acid resistance’) or combination (‘double resistance’, i.e. multi-resistance to two antibiotics) selective media; ‘mixed resistance’ refers to growth on both single-drug media, but not combination selective media. Initial mutator frequencies were 0.06 for ‘low’, 0.1 for ‘intermediate’ and 0.3 for ‘high’. MIC_WT_–minimum inhibitory concentration of the wild-type.

Fig 1 shows the number of populations with resistance in each treatment. Purely wild-type populations were capable of evolving single-drug resistance in single-drug environments, but were incapable of evolving double resistance in the combination treatment. The presence of mutators, and selection for resistance, were both required to observe the evolution of double resistance. In populations where mutators were introduced, but no antibiotics applied, only single-drug and mixed resistance evolved. However, when exposed to selection for resistance, a considerable proportion these populations evolved double resistance (23.3%–61.7%).

Using a Bayesian mixed-effects model (see S1 Appendix), we analysed the effects of mutator frequency and treatment on resistance at the end of the experiment (incorporating only main effects, as an interaction term did not significantly improve the fit; see Table A and Fig A in S1 Appendix). There was a positive association between the initial mutator frequency and the proportion of populations with double resistance, although with overlapping 95% C.I.s [low = 5.41 (3.57, 7.39), intermediate = 5.98 (4.36, 8.26), high = 8.01 (6.35, 10.28)]. The combination treatment was most effective overall at suppressing resistance. Nonetheless, we were surprised to find that double resistance was more likely to emerge in the single-drug treatments than the combination treatment [rifampicin = 7.80 (6.21, 9.97), nalidixic acid = 5.20 (3.71, 7.36), combination = 4.22 (2.78, 6.33)].

### Phenotypic and genomic characterisation of evolved multi-resistant isolates arising during ramping selection experiment

**Fig 2:**
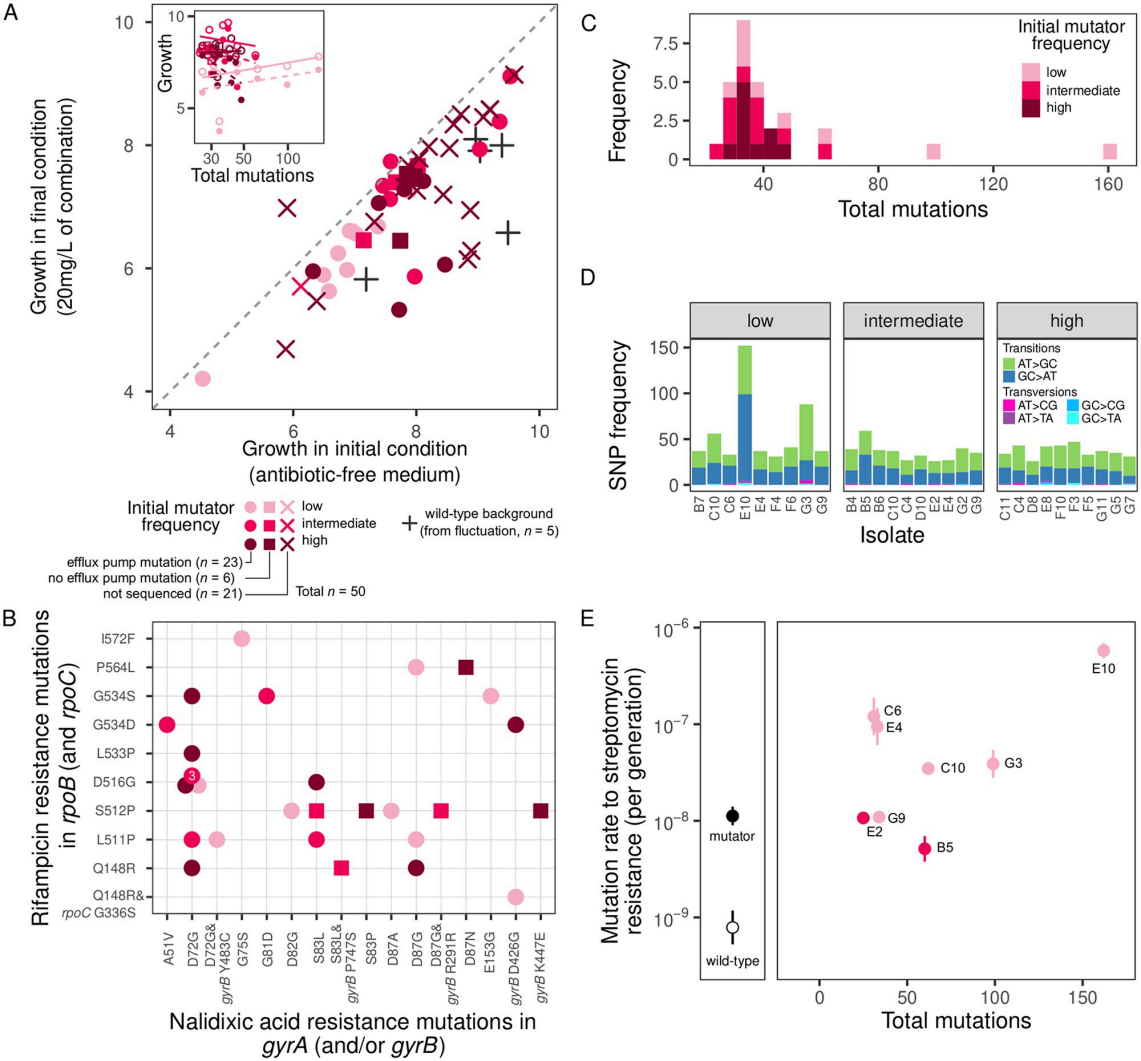
Phenotypic and genomic changes underpinning multi-resistance evolution in mutators. A. Relationship between growth of multi-resistant mutants under initial and final conditions of the selection experiment. Inset shows the relationship between growth and number of acquired mutations for antibiotic-free medium (solid line, open points) and 20 mg/l of the combination treatment (dashed line, filled points). Growth is measured as area under the curve of OD 600 nm growth curves taken over 22 h. B. Mutations identified in canonical drug resistance targets for rifampicin and nalidixic acid resistance. C. Number of mutations identified in each sequenced multi-resistant isolate. D. Spectrum of spontaneous SNPs is dominated by transitions. E. Number of mutations detected by whole genome sequencing is positively associated with mutation rate in multi-resistant isolates (measured by fluctuation test to streptomycin resistance on *n* = 8 isolates, *p*- value = 0.013, *R*^2^ = 0.50).

We assayed growth phenotypes of evolved multi-resistant isolates. There was a strong correlation between growth in minimum and maximum concentrations of the combination treatment (i.e. 0 mg/l and 20 mg/l) (Fig 2A, Bayesian multivariate regression: *r* = 0.73, 95% C.I.: (0.57, 0.84), see Fig B and Table B in S1 Appendix). There was little effect of either initial mutator frequency or the number of acquired mutations on fitness of the evolved isolates, which may potentially be explained by counterbalancing effects of acquiring both deleterious and beneficial mutations.

We performed whole genome sequencing and variant calling for a subset of multi-resistant isolates that arose in the combination treatment (see S2 Appendix). We found mutations in genes related to rifampicin and nalidixic acid resistance, as well as multi-drug efflux pumps (Fig 2B, and Table A in S2 Appendix). All isolates acquired mutations in the gene encoding the target of rifampicin, RNA polymerase subunit *rpoB*, with one strain acquiring an additional mutation in *rpoC*. All isolates acquired mutations in the gene encoding the target of nalidixic acid, DNA gyrase: 23/29 isolates possessed a single mutation in the *gyrA* component, 3/29 possessed a single mutation in the *gyrB* component, and 3/29 acquired mutations in both *gyrA* and *gyrB*. Diverse SNPs were observed for *rpoB* and *gyrA*. There was a significant association between specific SNPs occurring in *rpoB* and *gyrA* (Fisher’s exact test, *p* = 0.04), which arose because the pair *rpoB* D516G and *gyrA* D72G occurred 5 times in total. Most strains (23/29) also acquired mutations in multi-drug efflux pump genes and/or their regulators, the most frequent being *acrR* (9/29), a repressor involved in the AcrAB-TolC system [51]. However, efflux pump mutations included both synonymous substitutions and putative loss-of-function mutations in structural components, which are unlikely to improve resistance.

In addition to mutations the canonical targets of both antibiotics, we detected mutations across the genome (median = 34, range = 25–162, Fig 2C). The mutational spectrum of SNPs was dominated by transitions (Fig 2D), which is characteristic of the specific Δ*mutS* mutator allele introduced here [52, 53]. While some of this additional variation likely affected fitness, there was little association between the number of mutations identified through whole genome sequencing and growth (Fig 2A). Many mutations observed were likely to be selectively neutral, as 283/1252 (22.6%) of all mutations detected were synonymous SNPs. There was a negative association between mutator frequency and number of mutations acquired, although the estimated 95% C.I. of the slope overlapped with zero [Bayesian linear regression slope = −49.93, 95% C.I. = (−108.78, 8.53)]. Notably, we identified exceptionally high numbers of mutations in 4/29 isolates (three isolates with 62, 99, and 162 mutations each from the ‘low’ treatment, and one isolate with 60 mutations from the ‘intermediate’ treatment). We therefore assessed whether mutation rate itself had evolved during the experiment by measuring the mutation rates of eight multi-resistant isolates (the four mentioned above and four matched isolates from the same treatments), in comparison with the original Δ*mutS* strain and the wild-type. There was a positive association between estimated mutation rate and number of mutations each isolate had acquired during selection (Fig 2E). While three evolved isolates had estimated mutation rates approximately equal to (or slightly lower than) Δ*mutS*, five had increased mutation rates, ranging from 3-fold to 51-fold greater than Δ*mutS*—among most of these, we detected mutations in DNA replication and repair genes (Table B in S2 Appendix).

### Stochastic simulations reveal key mechanisms of multi-resistance evolution

To gain insight into the drivers of multi-resistance evolution, including the requirement for different classes of mutations observed, we used a stochastic population-dynamic simulation model. We produced a minimal model capable of predicting multi-resistance evolution, i.e. we included only a single allele for each resistance type, and excluded multi-drug resistance mechanisms, spontaneous double mutants, recombination, and mutations at non-resistance-conferring loci. Disallowing these types of variation permitted us to test hypotheses regarding the roles of selection and mutation supply on the emergence of multi-resistance. Populations initially contained only sensitive individuals, which we denote *S* for the wild-type (and *S*′ for the mutator, if present, differing only in mutation rate from the wild-type). During growth, single mutations can occur, giving rise to rifampicin-resistant type *R* (and *R*′) and nalidixic acid resistant type *N* (and *N*′). Subsequently, *R* and *N* (and *R*′ and *N*′) can each give rise to multi-resistant type *D* (and *D*′). We used a stochastic approach because it more accurately captures the dynamics of mutations that arise in a single cell, i.e. they occur as random events [54–56]. For each set of conditions, we simulated 1000 populations with a maximum population size of 5.71 × 10^8^ bacteria, equivalent to the maximum density observed in the selection experiment. Simulation parameters relating to growth were estimated empirically from growth curve data using strains that were derived from fluctuation tests and therefore independent from the selection experiment (Fig D in S3 Appendix; see S3 Appendix for full details on the curve fitting procedure). Mutation rates of our wild-type and mutator strains were estimated in previous work [24, 57].

**Fig 3:**
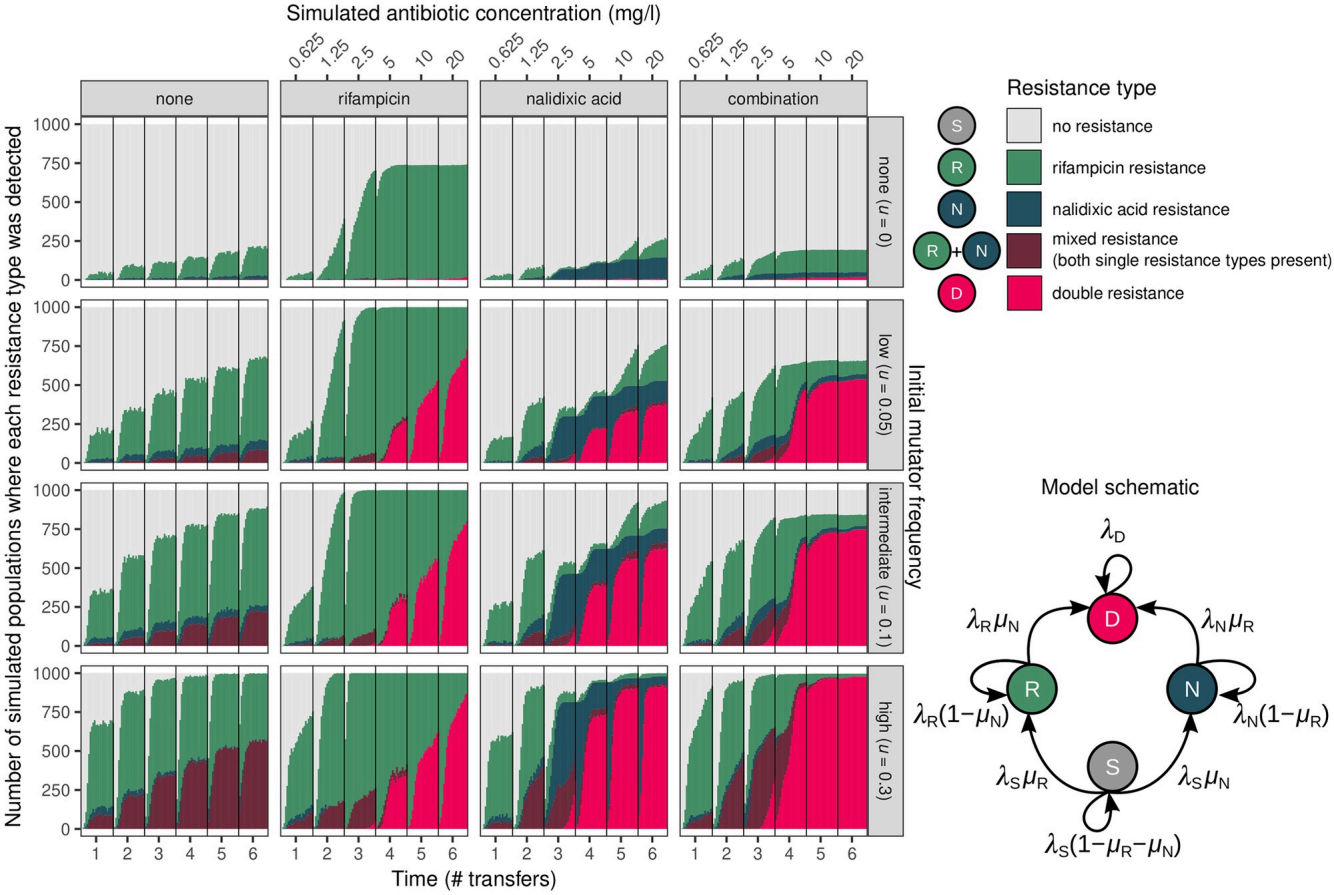
Simulated resistance evolution allowing only sequential acquisition of resistance recapitulated the emergence of resistance in experiments, suggesting elevated single-drug resistance mutation rates are sufficient to explain multi-resistance evolution. Resistance type was determined in an equivalent manner to experimental data in Fig 1 (i.e. if at least on individual of each type was detected in a random sample of 1/200 of each population, see Methods). The schematic depicts the the simulation approach, where λ_*i*_ refers to the reproduction rate of Type *i*, and *μ*_*j*_ refers to the mutation rate to resist antibiotic *j* (see S3 Appendix for full details). Parameters used in simulations are shown in Fig D in S3 Appendix.

This simulation model captured the major features of the wet-lab experiments (Fig 3, cf. Fig 1), i.e. that multi-resistance was constrained to populations treated with antibiotics, including single antibiotic treatments, and that the presence of mutators facilitated multi-resistance evolution. A Bayesian categorical model fitted to these simulations produced parameter estimates that closely matched those from the experimental data, demonstrating that the simulations quantitatively recapitulate the experiments (Fig H in S3 Appendix). For the nalidixic acid treatment, one notable difference was a higher probability of mixed resistance in the experiment than the simulation [experiment = 2.84 (2.18, 3.53) vs. simulation = −2.58 (−4.21, −1.21)]. This can be explained by the simulation overestimating transitions from mixed resistance to double resistance if there were additional costs of double resistance in the nalidixic acid environment that were not captured by our model (e.g. differences in lag time).

Note that a small fraction of simulated populations consisting only of wild-type individuals evolved multi-resistance (between 0/1000 and 15/1000, depending on treatment); this is consistent with expectations from our experimental results (< 1/60 per treatment). As the simulation excludes the possibility of multi-resistance emerging through a single reproductive event, this suggests that multi-resistance can emerge without invoking multi-drug resistance mechanisms (e.g. efflux pumps), simultaneous acquisition of two resistance mutations, or recombination.

**Fig 4:**
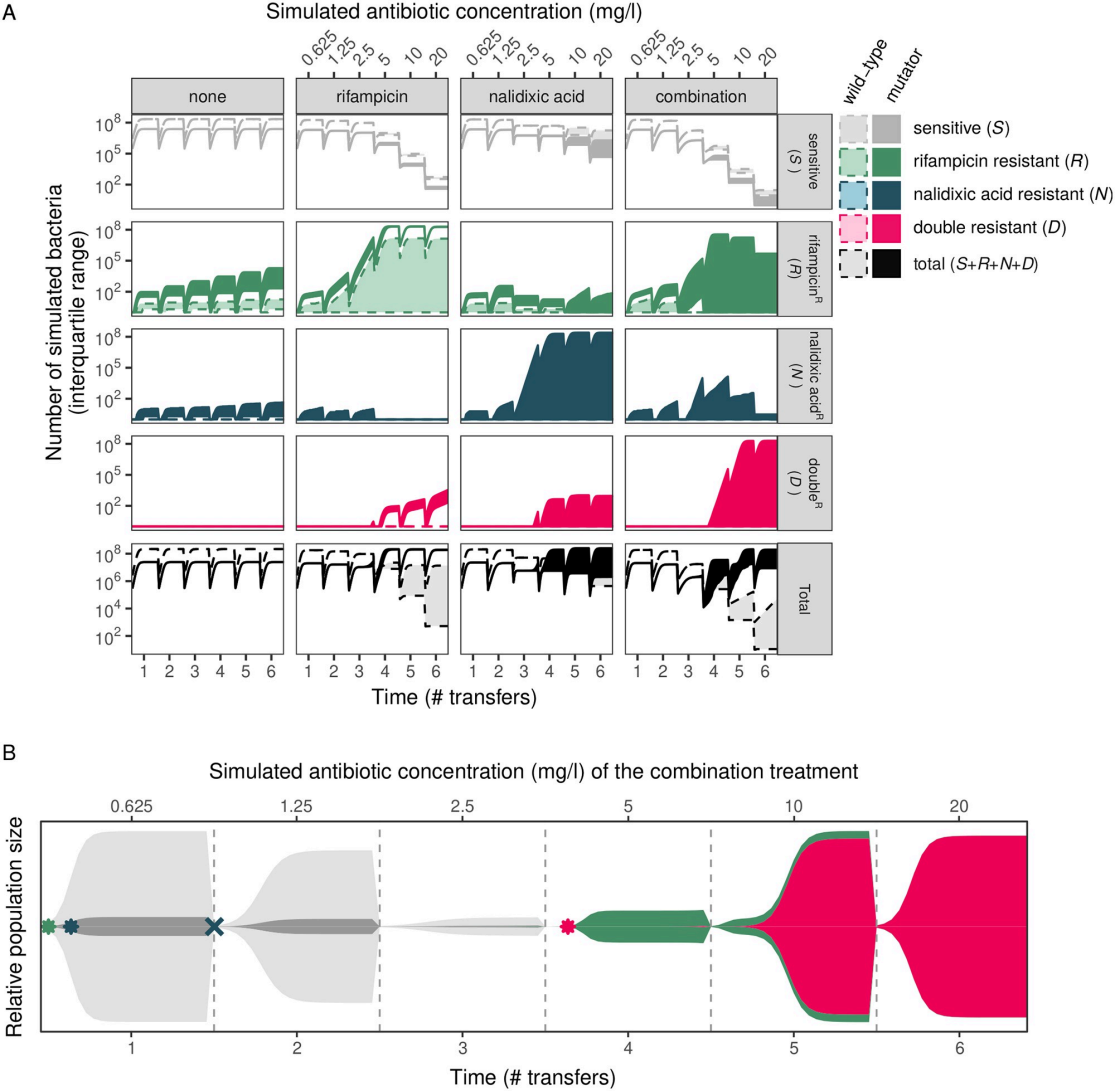
Population dynamics of simulated resistance evolution. A. Number of bacteria of each resistant type for the four simulated treatments (interquartile range, IQR, over *n* = 1000 replicate simulations). Results shown from the ‘intermediate’ initial mutator frequency (*u* = 0.1; other frequencies are shown in S1 Fig). B. Muller diagram showing typical progression from sensitive to multi-resistant in the combination treatment within a single population. In the combination treatment, multi-resistance most commonly emerged via *S*′ → *R*′ → *D*′ in the mutator genetic background (examples of other paths are shown in S2 Fig). Height of each area corresponds to the population size of each type. Example shown is a single replicate from the ‘intermediate’ initial mutator frequency (*u* = 0.1) treatment.

Simulations enabled us to observe the dynamics of multi-resistance evolution. Fig 4A and S1 Fig demonstrate that, while selective sweeps for single-resistance were observed in all antibiotic treatments, multi-resistance only swept to fixation in the combination treatment. Moreover, although the combination treatment was more effective at preventing the emergence of multi-resistance, once it had emerged, it facilitated the sweep of multi-resistance toward fixation. Within populations, Fig 4B shows the most commonly observed evolutionary trajectory to multi-resistance, *S*′ → *R*′ → *D*′ (examples of other trajectories can be found in S2 Fig). In general, single resistance arose early in the mutator genetic background, but remained at low frequency until it conferred a fitness benefit over the sensitive type. Once the single-resistant lineage began to increase in frequency, a subsequent mutation producing the multi-resistant type emerged. In some cases, resistant types can be lost due to population bottlenecks (as is the case for *N*′ in Fig 4B and *R*′ and *D*′ in S2 Fig); these may re-emerge and go on to found a *D*′ type lineage, or ultimately go extinct. *R*′ and *N*′ types can sometimes emerge and co-exit without going extinct, ultimately leading to two independent lineages resulting in *D*′ type individuals.

### Simulating other conditions

To evaluate the generality of these findings with regard to mutators, we ran further simulations with different parameter values. We first explored different dose escalation schemes, varying the amount of time until inhibition is achieved. We found that the presence of mutators facilitates multi-resistance evolution across a range of dose-escalation schemes (Fig I in S3 Appendix).

We next assessed whether our findings are specific to the particular empirical parameters we estimated (Fig D in S3 Appendix). We used parameter values that produce logistic growth with an imposed ‘cost of resistance’ under the antibiotic-free condition [58]. Even with these parameter values, we observed the evolution and spread of multi-resistance under the same conditions as occurred using our empirical estimates (Fig J in S3 Appendix).

Considerable attention has been given to how interactions between pairs of antibiotic can improve (‘synergistic’) or worsen (‘antagonistic’) their efficacy when used in combination [3, 13, 17–19, 59]. Synergistic combinations have been found to enhance resistance evolution, and antagonistic pairs to suppress it [13, 19]; our simulations concur with these findings, and also suggest that mutators may allow populations to overcome the ability for antagonistic combinations to suppress resistance evolution, to some extent (Fig K in S3 Appendix).

Finally, we explored whether large population sizes could allow multi-resistance evolution in the absence of mutators. We found that, in the combination treatment, population sizes > 5.71 × 10^10^ evolve multi-resistance in the absence of mutators at an appreciable rate (Fig L in S3 Appendix), although this represents a population size much larger than most typical infections [60–63].

## Discussion

A major motivation behind using antibiotic combination therapy is its presumed resilience against resistance evolution. We find that combination treatment can be effective at suppressing resistance evolution. However, our results question its resilience under two common scenarios that occur in clinic and nature: delayed inhibition and the presence of mutators. Multi-resistance evolved under both single-drug and combination treatments when mutators were introduced at frequencies often found in infection (Fig 1), despite conferring no clear advantage in single-drug environments. Increased mutation rate brought on by defective mismatch repair was sufficient to explain the emergence of multi-resistance via sequential acquisition of independent resistance mutations. Although other evolutionary mechanisms could be relevant in other contexts, they need not be invoked here, e.g. differences in birth and death rates affecting the emergence of resistance [64], acquiring fitter resistance alleles through clonal interference [65], differential supply of compensatory mutations [66], genomic variation influencing MIC [67], or co-evolution between genome and resistance mechanisms [68]. Our findings raise concerns about the effectiveness of combination treatment in combating the evolution of drug resistance. Due consideration should be given to the evolutionary consequences of combination therapy as mutators are frequently present in infections—particularly in chronic infections, for which combination therapies are especially important.

To reveal the key evolutionary mechanisms that allow mutators to evolve multi-resistance, we first characterised the phenotypic and genomic changes that occurred in isolates that evolved resistance to both antibiotics (Fig 2). All sequenced isolates had acquired mutations in the drug targets of rifampicin (*rpoB*) and nalidixic acid (*gyrA* and/or *gyrB*). Most isolates also acquired mutations in multi-drug efflux pumps, including loss of function mutations likely to increase expression, which is consistent with findings in other organisms subjected to combination treatment [69]. In our system, efflux pump mutations were not found in all sequenced isolates, and were never found without canonical resistance mutations. This demonstrates they were not strictly required to achieve multi-resistance here. However, efflux pump overexpression may allow additional time for canonical mutations to occur [67], or provide an additional fitness benefit to strains with canonical resistance mutations. In addition, isolates also possessed remarkable genomic diversity without any direct association with resistance. Much of this variation is likely selectively neutral as there was no association between numbers of mutations acquired and fitness (antibiotic-free: *r* = −0.01, combination: *r* = −0.06, Fig 2) and many observed mutations were synonymous SNPs. However, there is evidence that mutation rates themselves evolved. For some isolates, we found a 3- to 51-fold increase in mutation rate relative to the ancestral Δ*mutS* strain. We found mutations in genes associated with DNA replication and repair, which likely hitch-hiked alongside multi-resistance as it swept toward fixation. Selection for multi-resistance via mutator alleles therefore appears to come at little cost to the organism, while also sometimes modifying genes associated with multi-drug resistance (efflux pumps) and increasing the evolutionary potential for developing resistance to other antibiotics (via selective sweeps of mutation rate-modifying alleles). These unintended consequences of combination therapy are alarming, and suggest that thoughtful consideration of the population genetics of multi-resistance need consideration before combination therapies are deployed more broadly.

To determine which processes were essential for the evolution of multi-resistance, we carried out *in silico* stochastic simulations (Figs 3 and 4). This revealed that multi-resistance could arise via acquiring two independent resistance mutations. The simulations closely matched the experimental results, suggesting that, in spite of other variation observed, the original mutator allele was sufficient to enable multi-resistance evolution. This provides further evidence that the multi-drug efflux pump alterations and mutation rate increases seen in the selection experiment were not required for multi-resistance evolution in this instance (although efflux pumps can confer increased resistance evolvability [67]). In addition, the simulations also allowed us to delve into the evolutionary dynamics of multi-resistance occurring in individual populations. In all cases, mutator alleles hitch-hiked alongside single-drug resistance mutations, which then permitted a subsequent resistance mutation to occur in the same genetic background, which is consistent with previous work [70–73]. However, different antibiotic treatments had large differences in the frequency of multi-resistant individuals within the population: in the combination treatment, multi-resistance swept toward fixation, but achieved only low frequency in single-drug treatments where it confers little-to-no fitness benefit (Fig 4).

Mutators present other challenges for infection management beyond increasing the propensity for resistance, e.g. mitigating fitness costs of antibiotic resistance through compensatory adaptation [66], gaining resistance to non-antibiotic forms of bacterial control, such as vaccination [74] and phage therapy [75], and adapting to host conditions in opportunistic pathogens [76]. We found no evidence here that mutators suffered from diminished fitness (Fig 2A) due to the accumulation of deleterious mutations (i.e. ‘lethal mutagenesis’ or ‘mutational meltdown’ [77–79]), which is consistent with previous short-term [80] and long-term experiments [81] (excepting experiments where populations are kept artificially small, e.g. ref. [82]). Together, this suggests that, once multi-resistant mutator lineages become established, they will be difficult to eradicate—whether through natural selection or through alternatives to antibiotics. This raises the possibility that screening for mutators, in addition to antibiotic susceptibility, could be valuable in clinical practice.

Although our emphasis here was on mutators, our results are likely applicable to other factors that affect mutation rates, e.g. environmental factors such as nutrients and temperature [57, 83–86], stress-induced mutagenesis [87–90], radical-induced DNA damage [91, 92], and biotic interactions [24, 93–95]. Theoretical models have also predicted that increases in mutation rate *variability*, not just average, will lead to a higher probability of evolving multi-resistance [96]. A better understanding of these varied influences on mutation rates will be critical in the application of methods for preventing resistance through reducing the supply of resistance mutations [97–99].

In this study, we investigated the role of mutators in the evolution of multi-resistance using a simplified model system with two antibiotics. This allowed us to focus on the underlying mechanisms of mutator-driven resistance evolution. This approach cannot fully capture all of the diversity and heterogeneity encountered in real-world microbial populations. In particular, horizontally-acquired resistance mechanisms may considerably alter the evolutionary dynamics of the system [100]. Further, our experiments focused on a single drug combination, albeit one previously claimed to be robust against resistance [44]. While our additional simulation work suggests mutators will also facilitate resistance evolution to other combinations (Figs I–K in S3 Appendix), the extent to which mutators contribute to multi-resistance evolution more generally requires further assessment. For instance, simulations have shown that collateral sensitivity, where resistance to one antibiotic reduces the ability to tolerate another, may reduce the extent to which increases in mutation rate facilitate multi-resistance [101]. Finally, our system involved periodic population bottlenecks, and so may be largely applicable to populations with a high amount of population turnover (e.g. urinary tract infections via bladder voiding); whether our predictions hold where turnover is more gradual needs evaluation. More experimental and *in vivo* studies are therefore needed to confirm the generality of our results, and to determine the extent to which they apply to other microbial organisms and drug combinations.

## Conclusion

A vast number of combinations can be generated from existing antibiotics [102], which makes combination therapy an enticing approach for countering the rise of drug-resistant infections. While combinations treatments are indeed useful for reducing resistance evolution [40, 41], our results suggest that the potential for multi-resistance evolution needs thoughtful consideration in the design and application of such treatments. As direct assessment of all possible combinations is likely an insurmountable task [102], the experimental and modelling approaches developed here can serve as a framework for predicting whether particular combinations can suppress resistance evolution.

## Methods

### Strains and media

Selection experiments involved ‘wild-type’ *E. coli* str. K-12 substr. BW25113 [F-, Δ(*araD*-*araB*)567, Δ*lacZ*4787(::*rrnB*-3), λ-, *rph*-1, Δ(*rhaD*-*rhaB*)568, *hsdR*514] [103], and a ‘mutator’ strain Δ*mutS* (as above, but with Δ*mutS*738::kan, indicating Δ*mutS* replacement with kanamycin resistance). The kanamycin resistance cassette has not previously been observed to affect resistance to the antibiotics we have considered here [24, 57]. Both strains were obtained from Dharmacon, Horizon Discovery Group, UK. Relative to the published reference genome [104], whole genome resequencing revealed no pre-existing mutations in the wild-type BW25113 background, and a single point mutation in the Δ*mutS* strain (1,985,889 G>A, resulting in an amino acid substitution in *pgsA* A137V), which does not have a known association with resistance.

Routine culturing was performed in lysogeny broth [LB, 10 g/l tryptone (Fisher Scientific, UK), 5 g/l Bacto yeast extract (BD Biosciences, UK), 10 g/l NaCl (Fisher Scientific, UK)]. Selection experiments in the presence of antibiotic(s) were performed in Müller-Hinton broth (MH broth, 23 g/l, Sigma-Aldrich, UK). MH is a preferred medium for use with antibiotics [105]. Solid media were made by adding 12 g/l agar (BD Biosciences, UK) to either broth prior to autoclaving. Stock antibiotic solutions were prepared at 10 mg/ml. Rifampicin (Fisher Scientific, UK) was dissolved in methanol (Fisher Scientific, UK), and nalidixic acid (Fisher Scientific, UK) was dissolved in double distilled water, with 1N NaOH (Fisher Scientific, UK) added drop-wise until the antibiotic was solubilised. Strains were stored in LB with 40% glycerol at −80°C.

### Experimental evolution under single-drug and combination treatments

#### Establishment of mixed wild-type and mutator populations

We used experimental evolution to determine the effect of mutators on multi-resistance evolution under single and combination antibiotic treatments. Populations were founded from a mixture of mutator and wild-type individuals. Independent overnight cultures of wild-type and mutator were first grown separately in 5 ml MH broth. Volumetric mixtures of the mutator and wild-type overnight cultures were made at ratios of 0%/100% (‘none’), 10%/90% (‘low’), 25%/75% (‘intermediate’), and 50%/50% (‘high’) reflecting mutator frequencies observed in host-associated populations [26, 28, 30, 31]. We measured the actual frequency of mutators (denoted *u*) by plating serial dilutions of the populations on LB with 100 mg/l kanamycin agar (mutator count) and on LB agar (total population count), which was on average *u* = 0.06 for ‘low’, 0.1 for ‘intermediate’ and 0.3 for ‘high’ initial frequencies. The initial mixtures were assayed for resistance to rifampicin or nalidixic acid by plating on MH agar supplemented with rifampicin (50 mg/l) or nalidixic acid (30 mg/l). Five cultures were discarded for having detectable rifampicin resistance at the beginning of the experiment; no cultures had detectable nalidixic acid resistance.

#### Single-drug and combination selection environments

We used a serial transfer protocol that exposed the mixed wild-type and mutator populations to increasing concentrations of antibiotics over a period of six days. Four antibiotic treatment regimes were used: no antibiotic, rifampicin only, nalidixic acid only, or rifampicin and nalidixic acid combined. Antibiotic concentrations were doubled each day over the course of six days (0.625, 1.25, 2.5, 5, 10, 20 mg/l of each individual antibiotic), where inhibition of the ancestral strain was achieved on day 5, a protocol often used in resistance evolution experiments [3, 106–108]. The concentrations reflect physiological concentrations achieved during treatment with rifampicin (maximum serum concentration 5–18 mg/l [109]) and nalidixic acid (maximum plasma concentration 1.8–30 mg/l [49]). We note that nalidixic acid itself is mutagenic (approximately two-fold increase at 10 mg/l [110]), but the effect is minor relative to Δ*mutS* deletion.

#### Growth conditions

Experiments were performed in 96-well microtitre plates (Nunc, Fisher Scientific, UK) in 200 μL volumes grown at 37°C with 200 rpm shaking in an Innova 42R Incubator (Eppendorf, United Kingdom) for 22 h growth periods (‘days’). Position of each antibiotic treatment within the plate was assigned using stratified randomization. Populations were initiated from the mixed cultures by diluting 1 μL of each into 200 μL of fresh culture using a 96-pin replicator (Boekel Scientific, Feasterville, PA, USA). At the end of each day, population bottlenecks were imposed by pin replicating 1 μL of each population into 200 μL of fresh growth medium. This experimental protocol allows for a maximum of ∼ 46 generations of evolution (i.e. six days × 7.64 doublings/day). However, in practice the number of generations achieved by non-resistant strains in the presence of antibiotic(s) is likely to be less, given reduced carrying capacity at higher antibiotic concentrations (see Fig D in S3 Appendix).

#### Detection and analysis of resistance

Following each transfer, we used a high-throughput resistance assay involving pin replicating 1 μL of overnight culture (equivalent to a random sample of 1/200th of the population) on MH agar in 120 mm square Petri dishes, either without antibiotic, or with antibiotics at concentrations above the MIC of the wild-type: rifampicin (50 mg/l), nalidixic acid (30 mg/l), or rifampicin and nalidixic acid combined (50 mg/l and 30 mg/l, respectively). Growth at these concentrations is indicative of mutations in the canonical resistance genes for these antibiotics in *E. coli*, *rpoB* and *gyrA*, respectively. Populations were determined to be one of five ‘resistance states’: ‘sensitive’ (growth only on non-selective plates), ‘rifampicin resistant’ or ‘nalidixic acid resistant’ (growth only one of the two single-drug plates), ‘mixed resistant’ (growth on both single-drug selective plates but not combination selective plates), and ‘multi-resistant’ (growth on combination selective plates, as well as single-drug selective plates). Note these outcomes refer to *establishment* of resistance, rather than *fixation*, i.e. the proportion of resistant individuals is > 0 and ≤ 1. We analysed the probability of observing each resistance type using a Bayesian categorical model, implemented in the brms package [111, 112] in R 3.5.3 [113], described in full in S1 Appendix.

### Growth parameters of single- and multi-resistant clones

To determine the effects of single and multi-resistance on growth parameters, we selected five nalidixic acid resistant and five rifampicin resistant clones arising from the wild-type BW25113 genetic background via fluctuation tests [114], using an established protocol [115]. Briefly, 1 ml LB cultures of *E. coli* K-12 BW25113 were grown overnight in 96-well deep-well plates. The entire volume of each culture was plated on MH agar supplemented with rifampicin (50 mg/l) or nalidixic acid (30 mg/l) in the wells of a 6-well plate (each well approximately 35 mm in diameter). These 6-well plates were incubated for 48 h. To select multi-resistant clones, we performed a second fluctuation test using resistant strains from the first, plating on the antibiotic to which they were not already resistant. Colonies were isolated from selective plates, grown overnight in LB medium, and then stored at −80°C.

Growth curves were generated by measuring optical density (OD) at 600 nm every 30 min for 45 h using a BMG FLUOstar OMEGA with Microplate Stacker (BMG Labtech, Ortenberg, Germany). Each clone was grown in duplicate at 37°C under each of the antibiotic concentrations experienced during the selection experiments (i.e. 0.625, 1.25, 2.5, 5, 10, 20 mg/l each of rifampicin and/or nalidixic acid). Cultures were initiated by first growing clones overnight in 200 μL MH broth, then diluted 1/200 into a total volume of 200 μL MH broth containing one or both antibiotic(s). The growth curves were used to estimate growth rate and carrying capacity parameters for the stochastic simulation model using a custom MATLAB script (see ‘Data availability’ statement). The fitting procedure is described in full in S3 Appendix. We estimate *r*_*i*_ and *k*_*i*_ for resistant strains, rather than a simple growth threshold (e.g. MIC) because it directly relates to the population growth, mutation and selection occurring in this system. Moreover, we estimated growth parameters for each concentration, instead of imposing mechanistic constrains across environments and concentrations, because it allows for a more generalisable result.

In addition to fitting growth curves, we also summarised growth curves into a single metric, area under the curve (AUC, Fig A in S3 Appendix), as the empirical growth curves did not follow a standard logistic shape (which we also account for in the simulations below). We calculated AUC using the SummarizeGrowth function from the R package growthcurver [116]. AUC incorporates all of lag phase, growth rate, and density, and highly repeatable. Comparing the effect of the single-drug and combination concentrations on AUC, the effects of the antibiotics appears to be additive. To determine whether multi-resistance conferred a benefit under single-drug treatments, we fit a Bayesian multivariate linear regression model of AUC of different strains in the presence of each treatment over all concentrations (described in full in the S1 Appendix).

Using the same protocol as above, growth curves for the multi-resistant clones that evolved during the selection experiment (all in the mutator genetic background) were measured in antibiotic-free medium, and in 20 mg/l of the combination treatment. The association between AUC, initial mutator frequency and treatment was analysed using a Bayesian multivariate regression model (described in full in S1 Appendix).

### Mutation rate estimates and the probability of spontaneous double mutants

Mutation rates to rifampicin resistance (*μ*_*R*_ = 6.7 × 10^−9^ per cell division) and nalidixic acid resistance (*μ*_*N*_ = 7.4 × 10^−10^ per cell division), were obtained for the these strains in a previous publication [57], as was the mutator effect of Δ*mutS* (80-fold increase relative to wild type) [24]. Given that each antibiotic has independent targets, we assume that mutation rates to rifampicin resistance and nalidixic acid resistance are equal for wild-type and single-resistant strains, and constant across environments.

As each resistance arises independently, we can estimate the probability of simultaneously acquiring both resistance mutations during a single replication event (i.e. a ‘spontaneous double mutant’) for the wild-type as the product of their mutation rates to resistance [1, 25], i.e. *μ*_*R*_*μ*_*N*_ = 5.0 × 10^−18^, and for mutators as *μ*_*R*_*μ*_*N*_ × 80^2^ = 3.2 × 10^−14^. We can use these probabilities to obtain a rough estimate for the probability of observing a spontaneous double mutant by simulating a fluctuation test using rflan() from the R package ‘flan’ [117]. We performed 10^6^ simulations using the same parameters as the selection experiment (i.e. 60 independent populations, days 1–4 permitting wild-type growth, maximum population size of 5.71 × 10^8^). For populations comprising only wild-type individuals, no spontaneous double mutants were observed in 10^6^ simulations. For populations comprising solely mutators, a spontaneous double mutant was observed in 4210 out of 10^6^ simulations. Hence, the probability of having observed a spontaneous double mutant in the experimental setup seems likely to be very low.

### Whole genome sequencing and mutation identification

Whole genome sequencing was performed as described in S2 Appendix. Briefly, sequencing was performed on thirty multi-resistant isolates from the combination treatment. Genome sequencing was performed by MicrobesNG (http://www.microbesng.com, Birmingham, UK) according to their protocols (provided in S2 Appendix). Trimmed reads were then aligned to a reference genome and variants called using the breseq 0.36.1 pipeline [118, also see https://github.com/barricklab/breseq/]. The reference genome used was the *E. coli* K-12 BW25113 genome [104, NCBI accession CP009273.1], with additional annotations for insertion (IS) element regions to improve the calling of mutations related to IS insertion (modified Genbank format file, S1 File). One genome sequence did not correspond to *E. coli* K-12 BW25113 and was therefore discarded, leaving *n* = 9 genomes for the ‘low’ mutator treatment and *n* = 10 genomes for the ‘intermediate’ and ‘high’ mutator treatments. Genes relating to drug efflux, drug uptake, and DNA replication and repair were identified using the Ecocyc database (https://ecocyc.org [119]).

### Fluctuation tests on evolved multi-resistant isolates

Fluctuation tests were performed as previously described in ref. [115]. Briefly, ancestral BW25113, Δ*mutS* and eight evolved multi-resistant strains (four with ≥ 60 mutations identified by whole genome sequencing, and four randomly selected) were streaked from −80°C stocks onto LB agar and incubated overnight at 37°C. One colony each was inoculated into 5 ml of MH broth and grown overnight at 37°C and 250 rpm shaking. Cultures were diluted to an OD of approximately 0.3, and subsequently diluted 1000-fold into MH broth. For each strain, 16 parallel cultures of 300 μL were pipetted into deep-well 96-well plates and grown for 24 h. Each parallel culture was then pipetted onto selective media, i.e. TA agar with 25 μg/L streptomycin. Resistant mutants (*m* counts) were counted after 48 h growth. For each isolate, final population sizes (*N*_*t*_ counts) were estimated from *n* = 3 randomly-selected parallel cultures by serial dilution (to 10^−6^) and plating on TA agar. Mutation rates were estimated from *m* and *N*_*t*_ counts using mutestim() from the R package ‘flan’ [117].

### Stochastic population dynamics model

We numerically simulated resistance evolution using a stochastic population dynamic model according to the schematic in Fig 3. Model variables and parameters are described in full in Table A in S3 Appendix. The model describes four types of resistance *i* ∈ {*S*, *R*, *N*, *D*}, where *S* is antibiotic sensitive, *R* is rifampicin resistant, *N* is nalidixic acid resistant, and *D* is multi-resistant. We write *i*′ to refer to a type *i* with a mutator background (Fig 3). Here we consider a mutator that only differs in mutation rate from the wild-type, hence all growth parameters for types *i* and *i*′ are equal. Simulated populations were initiated with 5.71 × 10^6^ sensitive individuals; this is our estimate of the starting population size in the experiments obtained by serial dilution plating. Of the initial population of sensitive individuals, a fraction *u* were designated mutators, Type *S*′ (*u* = 0, 0.05, 0.1, or 0.3, approximating the observed frequencies in the ‘none’, ‘low’, ‘medium’ and ‘high’ mutator treatments) and the remaining 1 − *u* were designated wild-type, Type *S* (i.e. non-mutators). Bacterial growth was modelled as a Yule-Furry process, which has been used in many studies to describe resistance evolution [120–123] (see S3 Appendix). Note that this is a pure-birth process; the model makes the assumption that death, e.g. due to senescence or antibiotics, is negligible relative to the effect of periodic population bottlenecks (see below). We additionally allowed the reproduction rate to depend on population density (which provides equivalent results to continuous-time Gillespie simulations, but with reduced computational time; see Fig F in S3 Appendix).

At the start of the simulation, there were no Type *R*/*R*′, *N*/*N*′, or *D*/*D*′ individuals. Single-resistant Types *R*/*R*′ and *N*/*N*′, individuals must initially arise by mutation in reproduction events of Type *S*/*S*′ individuals. Once single resistant types exist in the population, they may produce further such individuals by reproduction. In reproduction events of individuals of Types *R*/*R*′ or *N*/*N*′, a subsequent mutation can occur, producing Type *D*/*D*′ individuals. Type *D*/*D*′ individuals may also reproduce, but we do not include further mutations in the model. We write *μ*_*R*_/*μ*_*R*′_ for the probability with which an offspring acquires resistance to rifampicin by mutation, and *μ*_*N*_/*μ*_*N*′_ for the probability that the offspring acquires resistance to nalidixic acid. We exclude the possibility that both resistance mutations can be newly acquired in the same reproduction event (i.e. no multi-drug resistance mechanisms, and no double-mutation events, which we have shown in the preceding section to be rare).

As the experiment involved a serial transfer protocol, population bottlenecks of 1/200 every 22 h were also simulated. Other parameter values were set to match the experimental procedure (initial frequency of mutators, dilution, duration of experiment; see Table E in S3 Appendix). Parameter values for growth rates and carrying capacities were estimated from OD growth curves using strains that were derived in an experiment independent of the selection experiments (see Figs B and C in S3 Appendix). The relationship between optical density and colony forming units was used to convert between OD units and numbers of bacteria (see Fig E and Table D in S3 Appendix).

## Supporting information

S1 Appendix Bayesian statistical analysis methods. (PDF)

S2 Appendix Whole genome sequencing and mutation identification. (PDF)

S3 Appendix Stochastic model of resistance evolution. (PDF)

S1 File Modified *Escherichia coli* K-12 BW25113 reference genome with additional IS element annotations. (GB)

S2 File Results of breseq analysis performed on genomes of evolved multi-resistant clones from the combination treatment. (ZIP)

S1 Fig Population dynamics of simulated resistance evolution, demonstrating that multi-resistance swept toward fixation only in the simulated combination treatment. Interquartile range (IQR) of the number of bacteria of each resistance type over time for the four simulated treatments for *n* = 1000 replicate simulations. Areas indicate the interquartile range (25% and 75% quantiles) of the numbers of bacteria of each resistance type from *n* = 1000 replicate stochastic simulations. Panels A–D show different initial mutator frequencies: ‘none’ (*u* = 0), ‘low’ (*u* = 0.05), ‘intermediate’ (*u* = 0.1, as shown in main text Fig 4), ‘high’ (*u* = 0.3).
(PDF)

S2 Fig Examples of the four most common types of within-population progression from sensitive to multi-resistant in the combination treatment, demonstrating the emergence of resistance via sequential acquisition of single-drug resistance mutations. Dynamics are described as follows: A. Single resistance emerged, but failed to establish and ultimately no double resistance is observed. B. Rifampicin resistance establishes first, followed by double resistance (equivalent to main text Fig 4A). C. Nalidixic acid resistance establishes first, followed by double resistance. D. Rifampicin resistance and nalidixic acid resistance both established, followed by double resistance arising in both genetic backgrounds. Areas correspond to the population size of each type. Examples shown are individual replicates from the ‘intermediate’ initial mutator frequency (*u* = 0.1) treatment.
(PDF)
