# Supplementary material for: Mutators can drive the evolution of multi-resistance to antibiotics
Source: PLoS Genet. 2023 Jun 13;19(6):e1010791. doi: 10.1371/journal.pgen.1010791 (PMC10292718; doi:10.1371/journal.pgen.1010791)
Supplement: S1 Appendix — (PDF) [file pgen.1010791.s001.pdf]

Mutators can drive the evolution of multi-resistance to antibiotics
Supporting Information

Danna R. Gifford, Ernesto Berríos-Caro, Christine Joerres,
Marc Suñé, Jessica H. Forsyth, Anish Bhattacharyya,
Tobias Galla, and Christopher G. Knight

S1 Appendix

Bayesian statistical analysis methods

Detection of resistance during experimental evolution

Defining the statistical model

Here we ask whether the initial mutator frequency and antibiotic treatments had an effect on resistance evolution, and whether these effects interacted. We fitted a categorical regression statistical model (also called a 'multinomial logistic' model) to the data, to analyse how different initial mutator frequencies and antibiotic treatments affected which type of resistance was observed at the end of the experiment. Note that in this section, 'model' refers to statistical model, and not to the 'stochastic population dynamics model' introduced later.

To make our analysis explicit, we will briefly describe the statistical model here. We will refer to Y_i as the value of the ith observation, $x_{m,i}$ as the independent variables, and $\beta_{m,k}$ as the estimated coefficients. It is the coefficients $\beta_{m,k}$ that are of interest as they relate how different experimental conditions influence the probability of observing any given outcome. Categorical regression can be formulated as an extension of logistic regression. Let Y_i be a categorical variable that takes a value k from $\{1, 2, ..., K\}$, and the probability that Y_i has outcome k be $\mathrm{P}(Y_i = k)$. We use a linear predictor function to compute $\mathrm{P}(Y_i = k)$. For a statistical model considering M explanatory variables, this takes the form

$$f(k, i) = \beta_{0,k} + \beta_{1,k}x_{1,i} + \beta_{2,k}x_{2,i} + \ldots + \beta_{M,k}x_{M,i}, \quad \text{(S1)}$$

where $\beta_{m,k}$ is the regression coefficient associated with the mth explanatory variable and the kth outcome, and $\beta_{0,k}$ is the intercept associated with the kth outcome. This function can be

written more compactly using vector notation and taking the dot product, $f(k,i) = \vec{\beta}_k \cdot \vec{x}_i$, where $\vec{\beta}_k$ and $\vec{x}_i$ each have length $M+1$.

The reader may be familiar with binary logistic regression with $K = 2$ outcomes, usually with $k = 1$ defined as 'success' and $k = 0$ as 'failure'. The function $f(k,i)$ is linked to the probability of observing outcome k by taking the log of the odds-ratio of the two outcomes, i.e. the logit function.

If $p_i = \mathrm{P}(Y_i = 1)$,

$$\begin{aligned} \text{logit}(p_i) &= \log\left(\frac{p_i}{1-p_i}\right) = \vec{\beta}_1 \cdot \vec{X}_i \\ p_i &= \frac{e^{\vec{\beta}_1 \cdot \vec{X}_i}}{1 + e^{\vec{\beta}_1 \cdot \vec{X}_i}}, \end{aligned} \tag{S2}$$

where $\vec{X}_i$ is the vector of values taken by the explanatory variables $\vec{x}_i$ for the observation Y_i.

For $K > 2$ outcomes, the multinomial logit can be thought of as computing $K - 1$ independent logistic regression models with respect to a consistent reference level [1]. If K is chosen as the reference level, $\beta_{0,K}$ is defined as the 'intercept' and all other elements of $\vec{\beta}_K$ are equal to zero. This results in

$$\begin{aligned} \log \frac{\mathrm{P}(Y_i = 1)}{\mathrm{P}(Y_i = K)} &= \vec{\beta}_1 \cdot \vec{X}_i \\ \log \frac{\mathrm{P}(Y_i = 2)}{\mathrm{P}(Y_i = K)} &= \vec{\beta}_2 \cdot \vec{X}_i \\ &\cdots \\ \log \frac{\mathrm{P}(Y_i = K-1)}{\mathrm{P}(Y_i = K)} &= \vec{\beta}_{K-1} \cdot \vec{X}_i \ . \end{aligned} \tag{S3}$$

The fact that $\sum_{k=1}^{K} P(Y_i = k) = 1$ allows calculating $\mathrm{P}(Y_i = K) = 1/\left(1 + \sum_{k=1}^{K-1} e^{\vec{\beta}_k \cdot \vec{X}_i}\right)$, which can then be used to solve the other probabilities. For any outcome c, the general form of $\mathrm{P}(Y_i = c)$ is thus given as

$$\mathrm{P}(Y_i = c) = \frac{e^{\vec{\beta}_c \cdot \vec{X}_i}}{1 + \sum_{k=1}^{K-1} e^{\vec{\beta}_k \cdot \vec{X}_i}}, \tag{S4}$$

which can then be used to estimate the coefficients $\vec{\beta}_k$ through various methods. The particular method we used, Bayesian categorical regression, is described in the next section.

Fitting the statistical model and hypothesis testing using Bayesian categorical regression

In our particular analysis, Y_i represents the type of resistance observed, with five possible outcomes: 'no resistance', 'rifampicin resistance', 'nalidixic acid resistance', 'mixed resistance'

and ‘double resistance’. For ‘mixed resistance‘, populations grew on selective plates containing either rifampicin or nalidixic acid, but *not* on plates containing both rifampicin and nalidixic acid, whereas ‘double resistant’ populations grew on plates containing both antibiotics. We note that these outcomes refer to *detection* of resistance, rather than *fixation*, i.e. the frequency of resistant individuals within each population has a value between > 0 and ≤ 1.

The independent variables, $\vec{x}_i$, were the initial mutator frequency, the antibiotic treatment applied, which microtitre plate the population inhabited, and position within each microtitre plate. Initial mutator frequency was treated as a categorical predictor (with levels ‘zero’, ‘low’, ‘medium’ or ‘high’, using ‘zero’ as the reference level), as we have no *a priori* expectation of a linear relationship between the proportion of mutators and the estimated coefficient. Antibiotic treatment was a categorical predictor (with levels ‘no antibiotic’, ‘rifampicin’, ‘nalidixic acid’, or ‘combination’, using ‘no antibiotic’ as the reference level). Initial mutator frequency and antibiotic were each treated as a fixed effect,as variance estimates from random effects variables with fewer than five levels tends to be imprecise [2]. Plate number and position within each microtitre plate were treated as random effects.

Incorporating both fixed and random effects requires fitting a ‘mixed-effects model’ to the data. However, mixed-effects models for categorical data are not straightforward to fit using standard frequentist inference methods. While it is possible to fit such models in principle [3], there is not, to our knowledge, a readily-available software implementation. However, mixed-effects categorical models can be fit using recently-developed tools that use Bayesian inference methods [4, 5]. We provide an example below. In addition to the software availability, there are additional benefits of using a Bayesian inference approach [6].

To estimate the coefficients, $\vec{\beta}_k$, we fit a mixed-effects categorical model using `brm()` from the `brms` package [4, 5] in R [7]. To fit a categorical model, ‘family’ was set to ‘categorical’ (with the default link ‘logit’). To ensure convergence was achieved, we set `max_treedepth` $= 15$ and `adapt_delta` $= 0.99$. We ran four chains of 2000 iterations each, with 1000 burn-in iterations. To permit hypothesis testing on point estimates, samples from specified priors were drawn by setting `sample_prior="yes"`. The choice of priors was based on preliminary data, and is described in detail in a later section. Default values were used for other settings.

To evaluate whether the interaction between initial mutator frequency and antibiotic treatment was important, we compared the full model (i.e. main effects and interaction) to a model with a main-effects only using ‘Pareto-smoothed importance sampling leave-one-out cross-validation’ (PSIS-LOO [8]). Incorporating interactions effects into the model did not significantly improve fit (PSIS-LOO difference in fit: -2.6 ± 5.4 S.E.), hence we present estimates from the main effects model.

An example of the function call is shown below (full R scripts are also available, see ‘Data Availability’ statement in the main text).

```
# Control parameters and priors
priors = c(set_prior ("student_t(7, -5, 2.5)", class = "Intercept"),
      set_prior ("student_t(7, 0, 2.5)", class = "b"))
```

```
controls = list(adapt_delta = 0.99, max_treedepth = 15)
# Model calls
M1.full = state ~ (pmutS.text + antibiotic)^2 + (1|row) + (1|col)
M1.main = state ~ (pmutS.text + antibiotic)   + (1|row) + (1|col)

modelM1.full = brm(M1.full,
      family = categorical("logit"),
      chains = 4, cores = 4, iter = 2000, warmup = 1000,
      prior = priors, control = controls, sample_prior = "yes",
      data = popnsday6)

modelM1.main = brm(M1.main,
      family = categorical("logit"),
      chains = 4, cores = 4, iter = 2000, warmup = 1000,
      prior = priors, control = controls, sample_prior = "yes",
      data = popnsday6)
```

Establishing priors for the model

Priors were established through a preliminary experiment in which pure populations of either wild-type or mutator bacteria were subjected to increasing concentrations of single and combination antibiotic treatments (see Methods in the main text). For this preliminary experiment, we assayed the proportion of populations by measuring OD at 600 nm relative to a 'blank' well with no bacteria present, using a BMG POLARstar OPTIMA plate reader (BMG Labtech, Ortenberg, Germany). A population was considered to be 'alive' with OD > 0.1, otherwise 'extinct'. The data are presented in Fig A. We observed that, in the absence antibiotic treatment, all populations survived. For single antibiotic treatments, all mutator populations survived, but some wild-type populations went extinct. For the combination antibiotic treatment, 0/54 wild-type and 39/54 mutator populations survived. We use this information to first establish priors for the intercepts of the model (Fig A).

As we observed no alive wild-type populations in the combination treatment, we therefore have some confidence that the proportion of multi-resistant outcomes is then $p_{\text{double}} < 1/54$. Therefore, the intercept for multi-resistance should be less than $\text{logit}(1/54) \approx -3.97$. We therefore used a t-distribution with mean $\mu = -5$ and broad and heavy tails with standard deviation $\sigma = 2.5$, and degrees of freedom $\nu = 7$, to reflect uncertainty. This distribution covers both $p_{\text{double}} \approx 0$ in the left tail, and $p_{\text{double}} = 39/54$, i.e. the number of alive populations in pure mutator populations, in the right tail. Given that the probability of being single-drug resistant in the absence of antibiotic is likely greater than double resistant, intercepts for other resistance outcomes are likely covered by this distribution, and so we use the same prior for the single-resistance intercepts.

Next, we use this information to establish priors for the other coefficients. We observed that the presence of mutators increases survival in the presence of both single and combination

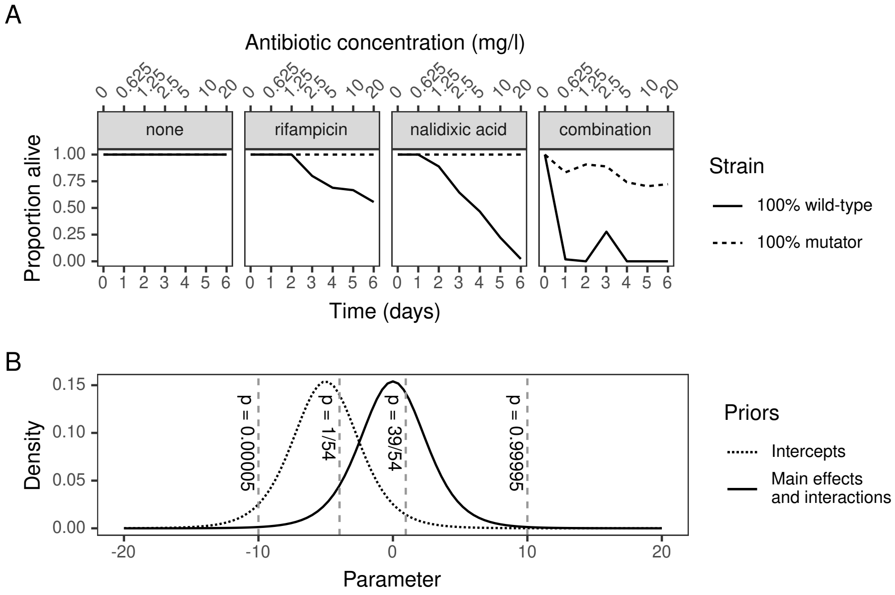

Fig A. Preliminary experiment with purely wild-type and mutator populations. A) The proportion of populations 'alive' (i.e. with OD > 0.1) under antibiotic treatments. B) Illustration of priors used to conduct Bayesian categorical regression using information from preliminary experiment, indicated by vertical dashed lines. Curves show the probability density for a Student's t distribution with means $\mu = 0$ (solid) or $\mu = -5$ (dotted), standard deviation $\sigma = 2.5$ (both curves) and degrees of freedom $\nu = 7$ (both curves).

antibiotics, hence the effect of mutators on *total* resistance (i.e. the sum of all resistance states observed) is likely to be positive. However, it is possible that different resistance states may have a negative relationship with the proportion of mutators, if for example, elevated mutation rates pushes double resistance to arise in the background of single-drug resistance, single-drug resistance may have a negative relationship with initial mutator frequency. Hence for the effects of initial proportion of mutators, we use a t-distribution centred at $\mu = 0$ with the same broad and heavy tails $\sigma = 2.5$, and $\nu = 7$ to reflect uncertainty. This covers the observed proportion of alive populations in a purely mutator population $p = 39/54$; we should however expect to observe fewer resistance events when the proportion of mutators is less than 1, as was the case in the selection experiment described in the main text. It also allows for the extreme possibilities of (nearly) zero or (nearly) all resistance, albeit with less weight given.

From this experiment, we have limited direct evidence for how the presence of antibiotics should affect the probability of observing resistance. On one hand, the presence of antibiotics decreased the number of alive populations (and an extinct population cannot be resistant). On the other, our growth measurements of resistant strains in the presence of antibiotics suggest a positive effect of being resistant in the presence of antibiotics, which would allow them to spread to high frequency and thus escape loss due to genetic drift. The effect of antibiotics is likely to be in the same range as for mutators (which includes 'no effect'), hence we use the same prior distribution (i.e. a t-distribution with $\mu = 0$, $\sigma = 2.5$, and $\nu = 7$).

Estimated model coefficients

Estimated model coefficients for the main-effects only model are shown in Table A. These are reported as treatment contrasts (i.e. relative to the treatment of no antibiotics and no mutators). We used 95% credible intervals (95% C.I.s) for all hypotheses tested. Coefficients were estimated on the logit scale (i.e. log-odds, which can assume any value between $-\infty$ and ∞, corresponding to proportions of outcomes of $p_{\text{resistance state}} = 0$ and 1 respectively). A coefficient is estimated for each possible combination of response outcome (resistance state) and the predictors (initial mutator frequency and antibiotic), which indicates how a specific combination of predictor levels (e.g. 'low' mutator frequency and 'rifampicin' treatment) influences the probability that a given resistance state is observed (e.g. 'rifampicin resistance').

Mutators generally have a positive effect on resistance, as indicated by positive treatment contrasts (Table A). However, as previously noted, although we expect an overall positive association between mutator frequency and resistance, we do not necessarily expect a straightforward relationship with each resistance state. This is because the 'mixed resistance' and 'double resistance' states follow from a single-drug resistance state. Indeed, this is the case for the 'low' mutator treatment and nalidixic acid resistance state, which is overtaken by 'mixed resistance'. Recall that the mixed resistance state contains both single-drug resistance types. Thus, to assess the effect of mutators on the presence of nalidixic acid resistance types, we can combine these treatment contrasts. We can perform Bayesian hypothesis tests on combinations of treatment contrasts using `hypothesis()`. The combined treatment contrast for

the ‘low’ mutator frequency for the combination of ‘nalidixic acid resistance’ and ‘mixed resistance’ is 3.36 [95% C.I. of (1.74, 5.00)], which is positive as predicted. The same combination of treatment contrasts could be performed for rifampicin resistance or for the other initial mutator frequencies, but as these already exclude zero, the combinations of their contrasts will also exclude zero.

Table A. Effects of initial mutator frequency and antibiotic treatment on resistance state observed at the end of the experiment. Estimated model coefficients come from fitting a Bayesian categorical regression model to the selection experiment outcomes. Treatment contrasts on the logit scale are shown. (* denotes 95% credible intervals excluding zero).

Resistance state	Coefficient	Treatment contrast	95% credible interval	
rifampicin resistance	intercept	-2.83	(-3.58, -2.14)	*
	low	1.48	(0.78, 2.25)	*
	intermediate	1.98	(1.23, 2.74)	*
	high	3.36	(2.56, 4.17)	*
	rifampicin	3.29	(2.60, 4.02)	*
	nalidixic acid	-1.22	(-2.28, -0.29)	*
	combination	-2.22	(-3.16, -1.38)	*
nalidixic acid resistance	intercept	-4.00	(-5.06, -3.08)	*
	low	-0.88	(-2.30, 0.34)	
	intermediate	1.51	(0.65, 2.39)	*
	high	2.81	(1.90, 3.78)	*
	rifampicin	-2.41	(-7.70, 0.75)	
	nalidixic acid	2.67	(1.88, 3.54)	*
	combination	-0.88	(-2.11, 0.26)	
mixed resistance	intercept	-6.78	(-8.46, -5.37)	*
	low	4.27	(3.02, 5.81)	*
	intermediate	4.89	(3.60, 6.45)	*
	high	6.51	(5.20, 8.10)	*
	rifampicin	3.63	(2.77, 4.52)	*
	nalidixic acid	2.84	(2.18, 3.53)	*
	combination	-0.96	(-1.90, -0.10)	*
double resistance	intercept	-10.99	(-13.91, -8.71)	*
	low	5.14	(3.57, 7.39)	*
	intermediate	5.98	(4.36, 8.26)	*
	high	8.01	(6.35, 10.28)	*
	rifampicin	7.80	(6.21, 9.97)	*
	nalidixic acid	5.20	(3.71, 7.36)	*
	combination	4.22	(2.78, 6.33)	*

Checking robustness against choice of priors

A potential consequence of using informative priors in Bayesian inference is that they may influence the posterior distribution for estimated coefficients unduly if they are not chosen appropriately. However, the use of non-informative priors does not necessarily mitigate these problems [for an in-depth discussion, see 9]. To check the robustness of our model against the originally chosen priors, we refit the model using different priors. We set Student-t priors on the intercept with arbitrarily chosen μ of -10, -20, -30, -40, with (as before) $\sigma = 2.5$ and $\nu = 7$. The estimated coefficients resulting from using these priors are quantitatively similar to our original model.

As a second approach to evaluating robustness, we also set strongly-informative priors on each coefficient of the model. We estimated means for each coefficient using a fixed-effects categorical model (i.e. without the random effects) by maximum likelihood using the `multinom()` function from the nnet package [10], which refers to this type of model as 'multinomial logistic regression'. Note that assigning priors in this fashion is used here only as a diagnostic technique, and is not recommended as a basis for assigning priors more generally. For the model with these strong priors, the majority of coefficients were again similar to the model with weaker priors. The exception was for coefficients associated with the 'double resistance' outcome. Here, because there were zero double resistance events associated with the reference levels of the main effects of the model (i.e. no mutators, no antibiotics), the maximum likelihood estimated the intercept associated with this outcome to be very small (log-odds of -38.34, equivalent to an odds ratio of approximately 4.5×10^{-16}). The posterior distribution for the intercept was dominated by this strong prior. Consequently, the estimated coefficients for the coefficients associated with antibiotic treatment and the presence of mutators where there were double resistant outcomes observed were much larger than those estimated when using the original weakly-informative priors. However, we note that in all cases, the qualitative outcomes with respect to antibiotic treatment and the presence of mutators remain unchanged.

Growth of strains derived from fluctuation tests

Defining the statistical model

Here we determined under which conditions resistant strains achieved a growth advantage (see Fig A in S3 Appendix). Growth was characterised using area under the curve (AUC) of growth curves from OD measured at 600 nm. We compare the wild-type (*E. coli* K-12 BW25113) with single- and double-drug resistant mutants selected in the BW25113 genetic background through fluctuation tests (see Methods in the main text). The model fitted to the data Fig 2 in the main text is a Bayesian two-way factorial model, with 'strain' and 'antibiotic' as predictor variables. We treated AUC measured in each antibiotic treatment as a multivariate response. This was on the basis that each strain was measured at several different concentrations, hence

may be non-independent. We used Student's t priors, as opposed to a Gaussian priors, because doing so is more robust against extreme values, i.e. values that appear to be 'outliers', but where there is no evidence of error in data collection [11].

Establishing priors for the statistical model

The intercept of this model is the mean AUC from the growth curves of OD, which is always greater than zero. To calculate empirical AUC, we used `SummariseGrowth()` from the `growthcurver`, which uses the trapezoidal rule to approximate the integral under the curve. OD values on this BMG FLUOstar OPTIMA plate reader are typically less than 1.2 for blank-corrected values. We used the trapezoidal rule to calculate extremes for the values of AUC, i.e.

$$\text{AUC} = \int_a^b f(x)dx \approx (b-a)\frac{f(a)+f(b)}{2}.$$

Since AUC was calculated over 25 h, the lower extreme is $(25-0)(0+0)/2 = 0$ (i.e. no growth) and the upper extreme is $(25-0)(1.2+1.2)/2 = 30$ (i.e. essentially instantaneous achievement of maximum density). However, previous experience from growing wild-type *E. coli* suggests they exit exponential growth in the region of 10 h after inoculation after a 1/1000 dilution in MH broth, giving

$$\text{AUC} \approx \frac{(10-0)(0+1.2)}{2} + \frac{(25-10)(1.2+1.2)}{2} = 24$$

as a rough approximation of what would be expected under good growth conditions. Hence, the prior for the intercepts should give highest density between 0 and 24. This was specified as a t-distribution with mean $\mu = 10$ and broad and heavy tails with standard deviation $\sigma = 2.5$, and degrees of freedom $\nu = 7$. The presence of mutators could either increase or decrease evolved fitness relative to the intercept. To set the priors on the effect of mutators, we use a t-distribution centred on mean $\mu = 0$, with standard deviation $\sigma = 0.5$, and degrees of freedom $\nu = 7$. If the intercept takes a value in the region of the mean of its prior, this prior on the mutator effect allows for the extreme possibility that the presence of mutators results in an AUC of zero (if the coefficient takes value -10), or an AUC beyond the technical capabilities of the equipment (for values > 10), with low probability. This is illustrated in Fig B.

Fitting the statistical model and hypothesis testing

As previously, model fitting was performed using `brm()`. To use a Student's t model, 'family' was set to 'student' (with the default link 'identity'). To permit hypothesis testing on point estimates, samples from specified priors were drawn by setting `sample_prior="yes"`. To ensure convergence, we set `max_treedepth` $= 15$ and `adapt_delta` $= 0.99$. Default values were used for other settings. As before, we used a 95% C.I. for hypotheses. As we are primarily interested in hypotheses on point estimates, we do not present all of the estimated effects here, though this can be generated with the R script provided (see the 'Data Availability' statement in

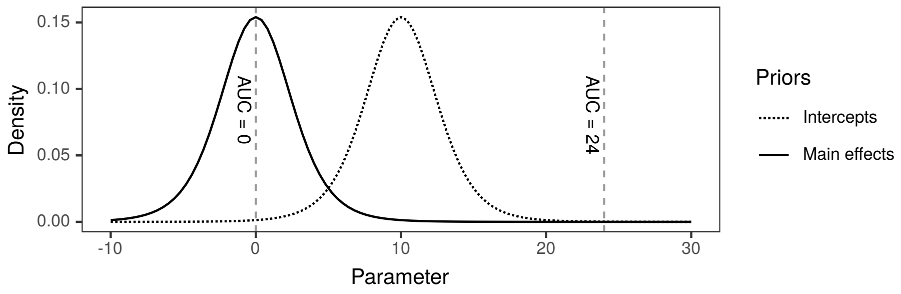

Fig B. Illustration of priors used to conduct Bayesian multivariate regression on the effect of mutators on growth. Curves show the probability density for a Student's t distribution with means $\mu = 0$ (solid) or $\mu = 10$ (dotted), standard deviation $\sigma = 2.5$ (both curves) and degrees of freedom $\nu = 7$ (both curves). Dashed vertical lines show prior information on technical upper and lower values for AUC, used to set a prior on the intercept.

the main text). We calculated the difference in AUC of the single-resistant and double-resistant strains in the two single-drug treatments. Estimated model coefficients are reported as treatment contrasts (i.e. relative to the wildtype and antibiotic-free treatment). For the rifampicin treatment, there was no benefit of double resistance over rifampicin resistance [95% C.I. of the difference = -0.07, 95% C.I.: $(-0.38, 0.23)$]. For the nalidixic acid treatment, there was a deleterious effect of double resistance over nalidixic acid resistance [95% C.I. of the difference = -0.75, 95% C.I.: $(-1.05, -0.45)$].

Growth of double resistant strains from selection experiment

Defining the statistical model

Here we determine whether initial mutator frequency had an effect on the growth of double resistant strains that evolved during selection. Decreased growth with a higher mutator frequency may occur if deleterious variation accumulated by the elevated mutation rate hitch-hikes to high frequency along with resistance. Alternatively, increased growth may be realised if the elevated mutation rate allowed the accumulation of beneficial mutations, or increased the clonal interference among resistance mutations. We fit a Bayesian multivariate Student's t mixed-effects model. We treated growth (measured by AUC) in the presence (20 mg/l) and absence (0 mg/l) as a bivariate response variable, and initial mutator frequency as the sole population-level factor ('low', 'intermediate', 'high', with the growth of double resistant strains derived in the BW25113 wild-type background as the reference level). As before, we used a bivariate model to account for correlations arising from measuring the AUC of each strain multiple times in different environments and a Student's t model to be robust to 'outliers' in the data. AUC at each concentration was measured over several 'replicate' experiments,

which was treated as a varying factor common to both response variables.

Establishing priors for the bivariate linear model

The same experimental procedure was used to measure growth of the selection experiment strains, hence we use the same priors as for the fluctuation test-derived strains (Fig B).

Fitting the statistical model and hypothesis testing

As previously, statistical model fitting was performed using `brm()`. To use a Student's t model, 'family' was set to 'student' (with the default link 'identity'). To permit hypothesis testing on point estimates, samples from specified priors were drawn by setting `sample_prior="yes"`. To ensure convergence, we set $\texttt{max\_treedepth} = 15$ and $\texttt{adapt\_delta} = 0.99$. Default values were used for other settings. As before, we used 95% C.I.s for hypotheses. Estimated coefficients are given in Table B, reported as treatment contrasts (i.e. relative to double resistance in the BW25113 strain). Growth measured by AUC in 0 mg/l and 20 mg/l of the combination antibiotic treatment was positively correlated [$r = 0.68$, 95% C.I.: $(0.56, 0.79)$]. The statistical model incorporating initial mutator frequency was a worse fit than an intercept-only model (WAIC 564.6 ± 23.7 SE vs. 540.0 ± 25.2 SE), suggesting initial mutator frequency did not have a large influence on AUC of double resistant strains.

References

[1] Becg CB, Gray R. Calculation of polychotomous logistic regression parameters using individualized regressions. Biometrika. 1984 Apr;71(1):11-8.

[2] Harrison XA. A comparison of observation-level random effect and Beta-Binomial models for modelling overdispersion in Binomial data in ecology & evolution. PeerJ. 2015;3:e1114.

[3] Hedeker D. A mixed-effects multinomial logistic regression model. Statistics in Medicine. 2003;22(9):1433-46.

[4] Bürkner PC. brms: An R Package for Bayesian Multilevel Models Using Stan. Journal of Statistical Software. 2017;80(1):1-28.

[5] Bürkner PC. Advanced Bayesian Multilevel Modeling with the R Package brms. The R Journal. 2018;10(1):395-411.

[6] Van Zyl CJJ. Frequentist and Bayesian inference: A conceptual primer. New Ideas in Psychology. 2018 Dec;51:44-9.

[7] R Core Team. R: A Language and Environment for Statistical Computing. Vienna, Austria; 2019. Available from: `https://www.R-project.org/`.

Table B. Estimated coefficients for the population-level effect of mutators on growth (measured by AUC) of multi-resistant clones in 0 mg/l and 20 mg/l of the combination treatment, from the fit of a Bayesian bivariate regression model. Treatment contrasts are shown (* denotes 95% credible intervals excluding zero).

Concentration	Coefficient	Estimate	Error	95% credible interval	
AUC in 0 mg/l	intercept	8.70	0.47	$(7.71, 9.58)$	*
	low	-1.68	0.59	$(-2.74, -0.43)$	*
	intermediate	-0.51	0.56	$(-1.55, 0.64)$	
	high	-0.58	0.53	$(-1.54, 0.53)$	
	σ(intercept)	-1.75	0.45	$(-2.60, -0.83)$	*
	σ(low)	0.57	0.50	$(-0.44, 1.53)$	
	σ(intermediate)	0.07	0.50	$(-0.95, 1.05)$	
	σ(high)	0.14	0.46	$(-0.82, 1.02)$	
AUC in 20 mg/l	intercept	7.20	0.46	$(6.27, 8.05)$	*
	low	-0.77	0.57	$(-1.85, 0.39)$	
	intermediate	0.17	0.55	$(-0.93, 1.26)$	
	high	-0.01	0.51	$(-0.94, 1.02)$	
	σ(intercept)	-2.13	0.48	$(-3.07, -1.12)$	*
	σ(low)	0.46	0.52	$(-0.61, 1.44)$	
	σ(intermediate)	0.41	0.53	$(-0.69, 1.43)$	
	σ(high)	0.39	0.48	$(-0.64, 1.30)$	

[8] Vehtari A, Gelman A, Gabry J. Practical Bayesian model evaluation using leave-one-out cross-validation and WAIC. Statistics and Computing. 2017;27(5):1413-32.

[9] Lemoine NP. Moving beyond noninformative priors: why and how to choose weakly informative priors in Bayesian analyses. Oikos. 2019;128(7):912-28.

[10] Venables WN, Ripley BD. Modern Applied Statistics with S. 4th ed. New York: Springer; 2002. ISBN 0-387-95457-0. Available from: `http://www.stats.ox.ac.uk/pub/MASS4`.

[11] Feng D, Baumgartner R, Svetnik V. A robust Bayesian estimate of the concordance correlation coefficient. Journal of Biopharmaceutical Statistics. 2015;25(3):490-507.
