# Supplementary material for: Mutators can drive the evolution of multi-resistance to antibiotics
Source: PLoS Genet. 2023 Jun 13;19(6):e1010791. doi: 10.1371/journal.pgen.1010791 (PMC10292718; doi:10.1371/journal.pgen.1010791)
Supplement: S2 Appendix — (PDF) [file pgen.1010791.s002.pdf]

Mutators can drive the evolution of multi-resistance to antibiotics
Supporting Information

Danna R. Gifford, Ernesto Berríos-Caro, Christine Joerres,
Marc Suñé, Jessica H. Forsyth, Anish Bhattacharyya,
Tobias Galla, and Christopher G. Knight

S2 Appendix

Whole genome sequencing and mutation identification

DNA extraction and library preparation

We performed whole genome sequencing on isolates from thirty of the evolved multi-resistant mutator populations. Genome sequencing was performed by MicrobesNG (`http://www.microbesng.com`, Birmingham, UK) according to the following protocols. Pure cultures of each strain were grown as a lawn covering approximately 1/3 of a 90mm Petri dish, with additional streaking on the remaining 2/3 to ensure culture purity. Cells were harvested and resuspended in DNA/RNA Shield (Zymo Research, USA). From each suspension, 5 to 40 µl was lysed with 120 µl of TE buffer containing lysozyme (final concentration 0.1 mg/mL) and RNase A (ITW Reagents, Barcelona, Spain) (final concentration 0.1 mg/mL), incubated for 25 min at 37°C. Proteinase K (VWR Chemicals, Ohio, USA; final concentration 0.1mg/mL). SDS (Sigma-Aldrich, Missouri, USA) (final concentration 0.5% v/v) was added and incubated for 5 min at 65°C. Genomic DNA was purified using an equal volume of SPRI beads (Beckman Coulter, USA) and resuspended in EB buffer (Qiagen, Germany). DNA is quantified with the Quant-iT dsDNA HS kit (ThermoFisher Scientific) assay in an Eppendorf AF2200 plate reader (Eppendorf UK Ltd, United Kingdom). Genomic DNA libraries were prepared using the Nextera XT Library Prep Kit (Illumina, San Diego, USA) following the manufacturer's protocol with the following modifications: input DNA is increased 2-fold, and PCR elongation time is increased to 45 s. DNA quantification and library preparation are carried out on a Hamilton Microlab STAR automated liquid handling system (Hamilton Bonaduz AG, Switzerland). Pooled libraries are quantified using the Kapa Biosystems Library Quantification Kit for Illumina. Libraries are

sequenced using Illumina sequencers (HiSeq/NovaSeq) using a 250bp paired end protocol. Adapters were trimmed from reads using Trimmomatic 0.30 with a sliding window quality cutoff of Q15 [1].

Sequence analysis and mutation calling

Trimmed reads were then aligned to a reference genome and variants called using the breseq 0.36.1 pipeline (`https://github.com/barricklab/breseq/`) [2]. The reference genome used was the *E. coli* K-12 BW25113 genome [3], with additional annotations for insertion (IS) element regions to improve the calling of mutations related to IS movement (modified Genbank available in as S1 File). One isolate was discarded after sequencing due to being the wrong organism, ultimately leaving for the analysis $n = 9$ genomes for the 'low' mutator treatment and $n = 10$ genomes for the 'intermediate' and 'high' mutator treatments. Mutations from all isolates were brought together into a single file using the breseq-provided utility program `gdtools ANNOTATE`. Mutations in canonical targets of resistance and efflux pumps, as well as the total number of mutations detected in each isolate, are given in Table A. Separate output files generated by breseq are provided as S2 File. Mutations relating to efflux pumps were identified using the Ecocyc database (`https://ecocyc.org`) [4].

We performed whole genome sequencing and variant calling on multi-resistant isolates. All 29 isolates acquired rifampicin resistance mutations in the canonical mutational target *rpoB*; for nalidixic acid resistance, 26/29 acquired mutations in the canonical target *gyrA*, and a further 3/29 in *gyrB*. Diverse mutations were observed in both of these targets (Table A), with the most prevalent being *rpoB* D516G and *gyrA* D72G. There was no particular association between specific SNP occurring in *rpoB* and *gyrA*, excepting the pair *rpoB* D516G and *gyrA* D72G, which occurred 5 times in total. In addition to mutations in these canonical targets, most strains (21/29) acquired a mutation in one of the multi-drug efflux pump systems in *E. coli*, the most frequently hit being *acrR* (9/29), a repressor involved in the AcrAB-TolC system. However, whether all such mutations are functionally significant is unclear, as these included both synonymous substitutions and putative loss-of-function mutations in structural components, which are not likely to improve efflux. In addition to resistance-associated mutations, we detected additional mutations across the genome (median = 34, range = 25–162, Fig 2C in the main text), with a mutational spectrum of SNPs consistent with Δ*mutS* (Fig 2D in the main text). While some likely affected fitness, it is likely that many mutations observed were selectively neutral, as 283/1252 (22.6%) of all mutations detected were synonymous SNPs. Notably, in 4/29 isolates, we identified a greater than average number of mutations (from the 'low' treatment: three isolates with 62, 99, and 162 mutations each, and from the 'intermediate' treatment: one isolate with 60 mutations). Among these, we detected mutations in DNA replication genes (Table B), indicating a potential advantage to an even higher mutation rate for acquiring multi-resistance.

Table A. Resistance-associated mutations detected in multi-resistant isolates that evolved via ramping selection in the combination treatment.

Mutator frequency	ID	Rifampicin resistance	Nalidixic acid resistance	Efflux mutations	Total mutations
low	B7	*rpoB* D516G	*gyrA* D72G	*acrR* A20V	39
	C6	*rpoB* S512P	*gyrA* D87A	*macB* C580Y, *mprA* R111C	31
	C10	*rpoB* Q148R, *rpoC* G336S	*gyrB* D426G	*acrB* G675G, *mprA* 125fs	62
	E10	*rpoB* P564L	*gyrA* D87G	*acrB* 23fs, *mdtK* W136R, *mdtE* R65R	162
	E4	*rpoB* L511P	*gyrA* D87G	*acrA* Q36X, *acrR* T44A, *emrK* D41G, *marR* G116D	33
	F4	*rpoB* S512P	*gyrA* D82G	between *acrA*/acrR [intergenic (-95/-47)]	26
	F6	*rpoB* G534S	*gyrA* E153G	*acrR* P206L, *marR* L97P, *mdtO* R542R	45
	G3	*rpoB* I572F	*gyrA* G75S	*acrA* V371A, *mprA* G121D	99
	G9	*rpoB* L511P	*gyrA* D72G, *gyrB* Y483C	*marR* A70T	34
intermediate	B4	*rpoB* L511P	*gyrA* D72G	*emrK* D41G, *marR* A70T, *mprA* L29P, *tolC* L234L, *rob* A70V	39
	B5	*rpoB* G534S	*gyrA* G81D	*emrK* D41G, *mprA* S84P, *mdtL* 186fs	60
	B6	*rpoB* G534D	*gyrA* A51V	*mprA* R111H	45
	C4	*rpoB* D516G	*gyrA* D72G	*acrR* 29fs, *marR* 126fs, *rpoS* K204E	29
	C10	*rpoB* D516G	*gyrA* D72G	*acrR* 189fs, *marR* 62fs	36
	D10	*rpoB* Q148R	*gyrA* S83L, *gyrB* P747S	none	32
	E2	*rpoB* L511P	*gyrA* S83L	*acrR* T44A	25
	E4	*rpoB* S512P	*gyrA* S83L	none	28
	G2	*rpoB* D516G	*gyrA* D72G	*acrR* R13H	37
	G9	*rpoB* S512P	*gyrA* D87G, *gyrB* R291R	none	30
high	C4	*rpoB* Q148R	*gyrA* D87G	*marR* R77H	40
	C11	*rpoB* G534D	*gyrB* D426G	*acrR* A41V, *emrK* D41G	32
	D8	*rpoB* D516G	*gyrA* S83L	*acrR* S172P	26
	E8	*rpoB* G534S	*gyrA* D72G	*marR* 62fs, *mprA* 97fs	41
	F3	*rpoB* Q148R	*gyrA* D72G	*acrR* 29fs	48
	F5	*rpoB* S512P	*gyrA* S83P	none	34
	F10	*rpoB* L533P	*gyrA* D72G	*mprA* L41P	44
	G5	*rpoB* P564L	*gyrA* D87N	none	31
	G7	*rpoB* S512P	*gyrB* K447E	none	31
	G11	*rpoB* D516G	*gyrA* D72G	*acrR* 189fs	33

X–mutation to premature stop codon; fs–frameshift mutation

Table B. Increases in mutation rate associated with mutations in known DNA replication and repair genes.

Mutator frequency	ID	Gene	Product	Mutation type	Total mutations	Fold change in μ
low	E10	*dnaK*	chaperone Hsp70, with co-chaperone DnaJ	S	162	51.1
		dnaQ	DNA polymerase III epsilon subunit	NS		
		umuD	DNA polymerase V, subunit D	S		
		recN	recombination and repair protein	NS		
		sbmC	DNA gyrase inhibitor	stop		
		ligA	DNA ligase, NAD(+)-dependent	S		
low	C6	—	—	—	31	11.5
low	E4	*polB*	DNA polymerase II	NS	33	9.07
low	G3	*nei*	endonuclease VIII	NS	99	3.57
		lon	DNA-binding ATP-dependent protease La	indel		
		mcrA	type IV methyl-directed restriction enzyme (e14 prophage)	NS		
		ung	uracil-DNA-glycosylase	S		
		smf	DNA recombination-mediator A family protein	NS		
low	C10	*dnaJ*	chaperone Hsp40, DnaK co-chaperone	NS	62	3.03
		polB	DNA polymerase II	indel		
low	G9	*cho*	endonuclease of nucleotide excision repair	NS	34	0.956
intermediate	E2	*nfo*	endonuclease IV with intrinsic 3'-5' exonuclease activity	NS	25	0.932
intermediate	B5	*uvrB*	excinulease of nucleotide excision repair	NS	60	0.467

NS–non-synonymous SNP; S–synonymous SNP; stop–premature stop codon; indel–insertion/deletion mutation

References

[1] Bolger AM, Lohse M, Usadel B. Trimmomatic: a flexible trimmer for Illumina sequence data. Bioinformatics. 2014 Aug;30(15):2114-20.

[2] Deatherage DE, Barrick JE. Identification of mutations in laboratory evolved microbes from next-generation sequencing data using breseq. Methods in Molecular Biology. 2014;1151:165.

[3] Grenier F, Matteau D, Baby V, Rodrigue S. Complete genome sequence of *Escherichia coli* BW25113. Genome Announcements. 2014;2(5):e01038-14.

[4] Keseler IM, Collado-Vides J, Santos-Zavaleta A, Peralta-Gil M, Gama-Castro S, Muñiz-Rascado L, et al. EcoCyc: a comprehensive database of *Escherichia coli* biology. Nucleic Acids Research. 2011 Jan;39(Database):issue.
