# Supplementary material for: Mutators can drive the evolution of multi-resistance to antibiotics
Source: PLoS Genet. 2023 Jun 13;19(6):e1010791. doi: 10.1371/journal.pgen.1010791 (PMC10292718; doi:10.1371/journal.pgen.1010791)
Supplement: S3 Appendix — (PDF) [file pgen.1010791.s003.pdf]

Mutators can drive the evolution of multi-resistance to antibiotics
Supporting Information

Danna R. Gifford, Ernesto Berríos-Caro, Christine Joerres,
Marc Suñé, Jessica H. Forsyth, Anish Bhattacharyya,
Tobias Galla, and Christopher G. Knight

S3 Appendix

Stochastic model of resistance evolution

Description of the model

We numerically simulated resistance evolution using a stochastic population dynamic model. A conceptual diagram of the model is shown in Fig 3 in the main text. The model describes four strains $i \in \{S, R, N, D\}$, where S is the sensitive ancestor, R is rifampicin resistant, N is nalidixic acid resistant, and D is double resistant.

Populations initially consist only of type S, a fraction u of which are mutators and $1 - u$ of which are wild-type. The conditions of the simulation are intended to replicate the selection experiment (see Methods in the main text for details). The simulated populations experience increasing antibiotic concentrations, which double each day. As in the experiment, populations are allowed to grow for a fixed time period, during which mutations can arise, before being subjected to dilution. These processes are described in the following sections.

Key variables and parameters are defined in Table A.

Yule–Furry process and negative binomial distribution

To simulate the growth of bacteria, we assume that each of the n_i individuals of strain i replicates at any time at a certain rate, regardless of its age. This rate will depend on the strain i, and on the composition of the population, as described below. For the time being we simply write λ_i for this rate. Setting mutations aside for the moment and assuming that replication

events are independent, this is described by the well-known Yule-Furry process, which has been used in many studies to describe resistance evolution [1–4]. Note that we assume that death due to senescence or the effects of antibiotics is negligible here, relative to the effect of periodic population bottlenecks (described in a subsequent section, 'Simulating the experiments').

The corresponding master equation reads

$$\dot{p}_{n_i}(t) = -n_i\lambda_i p_{n_i}(t) + (n_i - 1)\lambda_i p_{n_i-1}(t), \tag{S1}$$

where $p_{n_i}(t)$ is the probability that the number of bacteria of type i is n_i at time t. Here $\dot{p}_{n_i}$ represents the first derivative of the probability with respect to time. The first term on the right hand side of Eq. (S1) accounts for the outflux of probability (per unit time) of state n_i, and the second for the influx into state n_i. We denote the solutions of Eq. (S1) by $p_{n_i}(t; n_i^0)$ where n_i^0 indicates the number of bacteria at $t = 0$.

When the rate λ_i is constant in time the master equation Eq. (S1) can be solved analytically. The solution with initial condition $p_{n_i}(0; n_i^0) = \delta_{n_i,n_i^0}$ (where δ_{n_i,n_i^0} is the Kronecker delta) is the negative binomial distribution

$$p_{n_i}(t; n_i^0) = \binom{n_i - 1}{n_i - n_i^0} e^{-\lambda_i n_i^0 t}\left(1 - e^{-\lambda_i t}\right)^{(n_i - n_i^0)} = \binom{n_i - 1}{n_i - n_i^0} p_i^{n_i^0} q_i^{(n_i - n_i^0)}, \tag{S2}$$

where $p_i = e^{-\lambda_i t}$and $q_i = 1 - p_i$. The expression in Eq. (S2) is the probability that there are n_i individuals of strain i in the population at time t, given that there were n_i^0 such individuals at time $t = 0$. This does not involve any approximation for a pure birth process with constant reproduction rate λ_i.

Growth of bacteria

Saturated growth

In the following we write $n_T = n_S + n_R + n_N + n_D$ for the total number of bacteria in the population. In order to capture the saturation of growth found in the experiment we use the following expression for the reproduction rates λ_i for strain i when $n_T \le k_i$:

$$\lambda_i = \lambda_i(t) = r_i\left(1 - \frac{n_T}{k_i}\right). \tag{S3}$$

Given that n_T is time dependent, the reproduction rate $\lambda_i(t)$ is time dependent as well. The parameter k_i is the carrying capacity for strain i, and represents the maximum value n_i can take in absence of any other strain. The pre-factor r_i is the growth rate per unit time of strain i when there are very few individuals in the population (i.e., when the suppression of growth has not yet set in). The expression $r_i(1 - n_T/k_i)$ accounts for the interaction between the different strains.

Table A. Definitions of model variables and parameters.

Parameter	Definition
t	time (hours)
i	strain $i \in \{S, R, N, D\}$
n_i	number of individuals (of strain i)
$\bar{n}_i$	mean number of n_i over different realisations of the experiment
r_i	growth rate of strain i
k_i	carrying capacity of strain i
μ_j	mutation rate to resist antibiotic, $j \in \{R, N\}$
n_T	total population size, $n_T = \sum_i n_i$
$\bar{n}_T$	mean of n_T over different realisations of the experiment
Δt	length of time per simulation time-step
n^c	critical population size at growth regime switch
t_c	time at growth regime switch
u	initial frequency of mutators

Terms of this type are common in Lotka–Volterra type models. In writing down the above expression for $\lambda_i(t)$ we have assumed that the interaction is the same between each pair of strains (pure scramble competition, i.e. no direct interference, cross-feeding or similar).

We interpret the reproduction rate $\lambda_i(t)$ as an effective quantity, taking into account both birth and death processes of bacteria. We set the reproduction rate $\lambda_i(t)$ to zero when n_T exceeds k_i. This guarantees that the growth of strain i saturates when $n_T = k_i$, and at the same time it prevents the species with the highest carrying capacity from automatically taking over the population, as explained in more detail in ref. [5]. We note that this approach has implications for the stochastic dynamics. Birth death processes with the same net rate have very different outcomes depending on how 'birth' and 'death' are defined. For instance, if birth and death are both large in magnitude, $\lambda_i(t)$ may be zero but many more offspring will be produced, which has implications for the generation of mutations (discussed in a later section).

Connection to Lotka–Volterra dynamics

To make the connection to the well-known Lotka-Volterra dynamics more explicit, we note that the expected value of n_i obtained from the distribution in Eq. (S2) is $\bar{n}_i(t) = n_i^0 e^{\lambda_i(t)t}$. This means that the mean number of individuals $\bar{n}_i(t)$ at time t fulfills the relation $\mathrm{d}\bar{n}_i/\mathrm{d}t = \lambda_i(t)\bar{n}_i$. With the choice of $\lambda_i(t)$ as in Eq. (S3) this turns into a competitive Lotka-Volterra equation

$$\frac{\mathrm{d}\bar{n}_i}{\mathrm{d}t} = r_i \bar{n}_i \left(1 - \frac{\bar{n}_T}{k_i}\right). \tag{S4}$$

If we consider a a ‘pure culture’ where only a single type of bacterium is present, i.e. $\bar{n}_i = \bar{n}_T$, this has the analytical solution

$$\bar{n}_i(t) = \frac{k_i \bar{n}_i(t_0)}{\bar{n}_i(t_0) + [k_i - \bar{n}_i(t_0)]e^{-r_i(t-t_0)}} \quad . \tag{S5}$$

Eq. (S5) describes a sigmoidal dynamic, starting from $\bar{n}_i(t_0)$ at time t_0, and approaching an asymptotic limit at the carrying capacity k_i at long times. This is the familiar logistic growth model frequently employed in modelling of bacterial growth. We will use this form in Section S3 Appendix to estimate the growth parameters from experimental data for each type grown in pure culture.

Simulation method

Continuous-time birth processes can in principle be simulated using the Gillespie algorithm [6, 7]. This is an exact procedure for the production of sample paths. However, carrying out the Gillespie algorithm becomes time consuming when the populations are large. This is because the number of events occurring in the population per unit time is proportional to the size of the population. Bacterial populations often contain numbers of individuals of order 10^9; in the case of our simulations population sizes can go up to 5.71×10^8. This makes continuous-time Gillespie simulations unrealistic.

In such situations one therefore has to resort to approximations. One such approximation method is the so-called τ-leaping variant of Gillespie’s algorithm [8]. This approach proceeds in discrete time and assumes that total event rates in the population are constant over each step. The number of offspring produced in each step is then a Poissonian random number. Even in the case of constant reproduction rates $\lambda_i(t)$ this is no longer an exact procedure. Mathematically, the sampling is not from the true solution of the master equation [the negative binomial distribution in Eq. (S2)]. This is because the τ-leaping algorithm does not capture events in which an offspring generated in one step undergoes a further reproduction event in the time step, a sequence of processes which is in principle possible in the continuous-time model.

Like the τ-leaping method, our simulations proceed in discrete time, with a time step Δt. However we do not make the above Poissonian approximation, instead our sampling is from the negative binomial distribution [Eq. (S2)]. For a pure birth process this would mean to sample the number of individuals $n_i = n_i(t + \Delta t)$ at time $t + \Delta t$ from the distribution

$$p_{n_i}(t + \Delta t; n_i(t)) = \binom{n_i - 1}{n_i - n_i(t)} e^{-\lambda_i(t) n_i(t)\Delta t} \left(1 - e^{-\lambda_i(t)\Delta t}\right)^{n_i - n_i(t)}, \tag{S6}$$

where $n_i(t)$ is the number of individuals of strain i at time t, and where $\lambda_i(t)$ is the reproduction rate for this step. As discussed above and unlike τ-leaping, this is an exact procedure if the rate $\lambda_i(t)$ does not vary over the time step.

In our model the reproduction rates $\lambda_i(t)$ depend on the total number of individuals in the population, n_T. They therefore become time dependent, with interactions between the strains. The simulation method is then an approximation of the continuous-time process. We provide a comparison against simulations of the full continuous-time model further below.

Mutations

The simulation model described in the conceptual diagram in main text Fig 3 involves mutations. These can occur from sensitive type bacteria S to either types R or N, and, in turn, from type R and type N to type D. The model excludes the possibility of direct mutations from S (sensitive) to D (double resistance).

In order to incorporate mutations, we first calculate the number of offspring produced by a strain in a time step. Starting with $n_i^0 = n_i(t)$ cells of type i at the beginning of the time step we write $n_i(t + \Delta t)$ for the number of cells of strain i at the end of the step. The number of offspring m_i generated in this step is then

$$m_i = n_i(t + \Delta t) - n_i^0. \tag{S7}$$

Therefore, the probability Prob $(m_i = x) = P_{m_i}(t + \Delta t; n_i^0)$ that m_i offspring have been produced in the time step from t to $t + \Delta t$ is equal to the probability that there are $n_i(t) = n_i^0 + m_i$ individuals of strain i in the population at time $t + \Delta t$.

Using Eq. (S2) this results in the following expression for the distribution of m_i:

$$\text{Prob}\,(m_i = x) = P_{m_i}(t + \Delta t; n_i^0) = \binom{n_i^0 + x - 1}{x} p_i^{n_i^0} q_i^x. \tag{S8}$$

We assume that offspring of an individual of type S are of type R with probability μ_R or of type N with probability μ_N. With the remaining probability $1 - \mu_R - \mu_N$ the offspring of a parent of type S is also of type S (no mutation). Similarly, an offspring of a parent of type R is of type D with probability μ_N, and an offspring of a parent of type N is of type D with probability μ_R.

Summarising, the simulations proceed as follows:

1. Assuming that there are $n_i(t)$ individuals of strains $i \in \{S, R, N, D\}$ at time t, compute $\lambda_i(t)$ via Eq. (S3), and from this the $p_i = e^{-\lambda_i(t)\Delta t}$ and $q_i = 1 - p_i$.
2. Then draw the number of offspring m_i for each type from the distribution in Eq. (S8).
3. We first focus on the number offspring $m_S(t)$. The number of these that experience a mutation into type R is binomially distributed $n_{SR}(t) = \text{Binomial}[m_S(t), \mu_R]$. The number of offspring with mutations into type N follow $n_{SN}(t) = \text{Binomial}[m_S(t), \mu_N]$. The remaining offspring of a parent of type S is of type S, $n_{SS}(t) = m_S(t) - n_{SR}(t) - n_{SN}(t)$.

Similarly, the number of offspring of R with a mutation into type D will be $n_{RD}(t) = \text{Binomial}[m_R(t), \mu_N]$ and the remainder $n_{RR}(t) = m_R(t) - n_{RD}(t)$ is type R. Analogously, for strain N we have $n_{ND}(t) = \text{Binomial}[m_N(t), \mu_R]$ and $n_{NN}(t) = m_N(t) - n_{ND}$ for offspring of type D and N, respectively.

The number of individuals at the end of the time-step are then

$$\begin{aligned} n_S(t+\Delta t) &= n_S(t) + n_{SS}(t), \\ n_R(t+\Delta t) &= n_R(t) + n_{RR}(t) + n_{SR}(t), \\ n_N(t+\Delta t) &= n_N(t) + n_{NN}(t) + n_{SN}(t), \\ n_D(t+\Delta t) &= n_D(t) + m_D(t) + n_{RD}(t) + n_{ND}(t). \end{aligned} \tag{S9}$$

4. Increment time by Δt and go to step 1.

We have written $\text{Binomial}[n, p]$ for a binomial random number with parameters n and p; the probability that such a random number takes value j is $p_j = \frac{n!}{j!(n-j)!} p^j (1-p)^{n-j}$, for $j = 0, 1, \ldots, n$.

For the model described in Eq. (S9), we can represent the mutation pathways in matrix form for each time step $t \longrightarrow t + \Delta t$ as

$$\begin{aligned} \begin{bmatrix} n_S \\ n_R \\ n_N \\ n_D \end{bmatrix} (t+\Delta t) &= \begin{bmatrix} n_S \\ n_R \\ n_N \\ n_D \end{bmatrix} (t) + \begin{bmatrix} m_S(t) - n_{SR}(t) - n_{SN}(t) & 0 & 0 & 0 \\ n_{SR}(t) & m_R(t) - n_{RD}(t) & 0 & 0 \\ n_{SN}(t) & 0 & m_N(t) - n_{ND}(t) & 0 \\ 0 & n_{RD}(t) & n_{ND}(t) & m_D(t) \end{bmatrix} \begin{bmatrix} 1 \\ 1 \\ 1 \\ 1 \end{bmatrix} \\ &= \begin{bmatrix} n_S \\ n_R \\ n_N \\ n_D \end{bmatrix} (t) + \begin{bmatrix} m_S(t) - n_{SR}(t) - n_{SN}(t) \\ m_R(t) + n_{SR}(t) - n_{RD}(t) \\ m_N(t) + n_{SN}(t) - n_{ND}(t) \\ m_D(t) + n_{RD}(t) + n_{ND}(t) \end{bmatrix}. \end{aligned}$$

The periodic dilution in simulations of the laboratory experiments is implemented separately, for further details see S3 Appendix.

Estimating growth parameters from experimental data

We used experimental data to estimate values for the parameters r_i and k_i introduced in Eq. (S3). We carried out growth curve experiments of the wild-type sensitive strain (type S) and resistant strains (types R, N or D) by measuring optical density (OD) over time (Fig A), under conditions matching the selection experiment (see Methods in the main text). For each resistant type, we isolated five independent mutant clones derived from the wild-type S type via independent fluctuation tests (see Methods in the main text).

The growth curves deviate from a typical 'S'-shaped logistic curve, instead characterised by two 'plateaus', reminiscent of diauxic growth. Diauxic growth has been previously reported

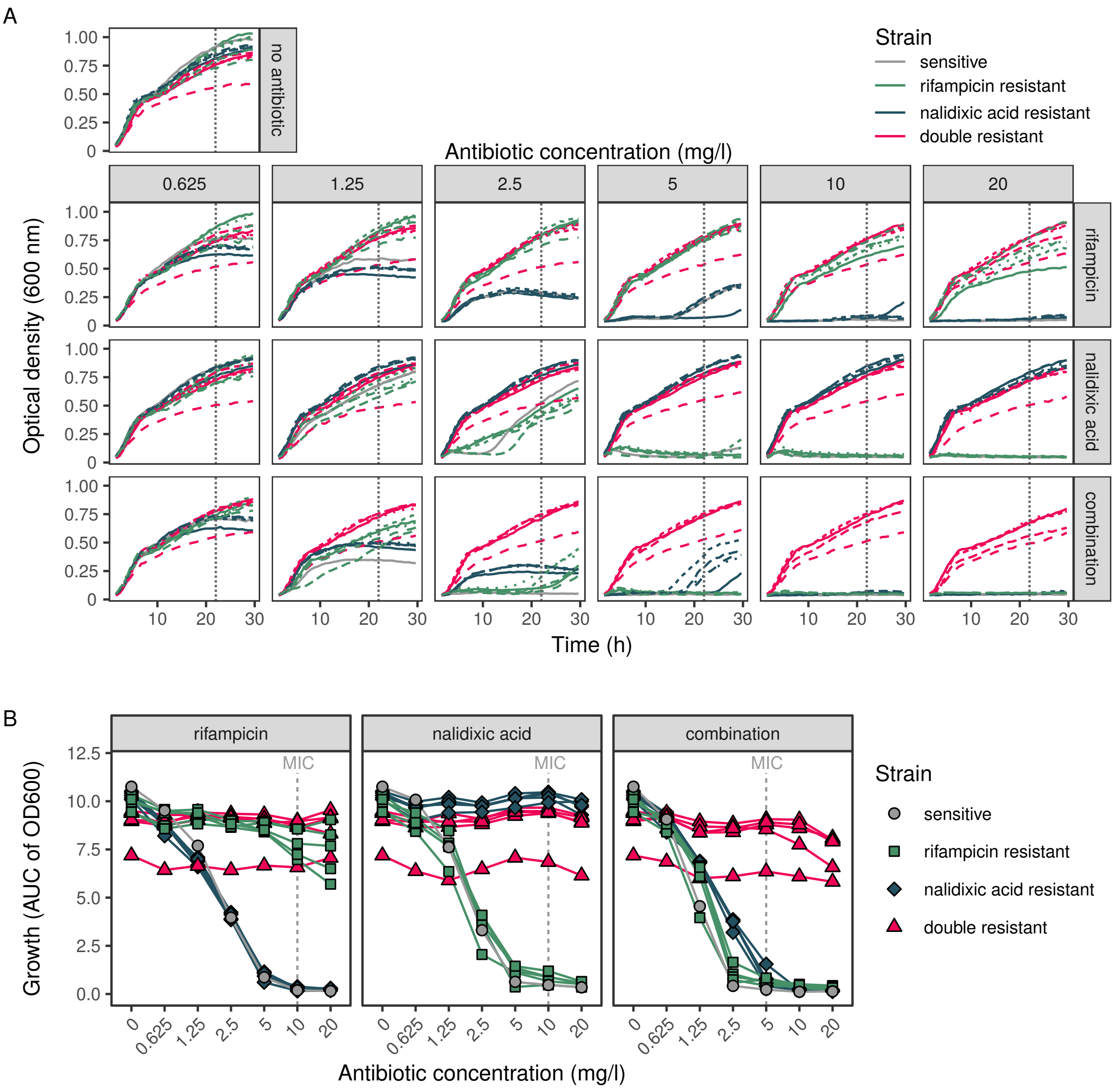

Fig A. Growth of sensitive, single-, and double-drug resistant strains under antibiotic concentrations used during the selection regime. Strains shown here were derived from the wild-type background, independently of the selection experiment. A) Growth curves, with each line depicting one of five different strains (indicated by line type), averaged over replicate growth curves ($n = 5$). Note curve shapes reminiscent of diauxic growth. B) Area under the the curve for each strain.

for MH media [9], as well as for other rich media [10, 11]. We account for this two-stage growth by fitting the data to growth curves of the form

$$n_i(t) = n_i^{(1)}(t)H(t_c - t) + n_i^{(2)}(t)H(t - t_c), \tag{S10}$$

where $n_i^{(1)}(t)$ and $n_i^{(2)}(t)$ are the same functionals as Eq. (S5):

$$n_i^{(1)}(t) = \frac{k_i^{(1)} n_i^c}{n_i^c + [k_i^{(1)} - n_i^c]e^{-r_i^{(1)}(t-t_i^c)}}, \quad n_i^{(2)}(t) = \frac{k_i^{(2)} n_i^c}{n_i^c + [k_i^{(2)} - n_i^c]e^{-r_i^{(2)}(t-t_i^c)}}, \tag{S11}$$

and where $H(t)$ is the Heaviside step function, $H(t) = 0$ for $t < 0$, $H(t = 0) = 1/2$ and $H(t) = 1$ for $t > 0$. $k_i^{(1)}$ and $k_i^{(2)}$ are the carrying capacities of strain i before and after the switch. The functions $n_i^{(1)}(t)$ and $n_i^{(2)}(t)$ are logistic growth curves that represent the two regimes of the diauxic growth. They are solutions of the one-strain logistic equation, Eq. (S5). The growth switches from $n_i^{(1)}$ to $n_i^{(2)}$ at time t_c, where $n_i^{(1)}(t)$ describes the growth for $t < t_i^c$ and $n_i^{(2)}(t)$ for $t > t_i^c$. Using the definition of the Heaviside function $H(t = 0) = 1/2$, one has $n_i(t) = n_i^c$ at $t = t_i^c$.

We illustrate the curve fitting for the rifampicin resistant type grown in the presence of the rifampicin, at the same concentrations used in the selection experiment (Fig B). For the purposes of the fit we only considered the first 25 h of the growth experiment, to eliminate potential complications introduced by evaporation of the growth medium. We measure time in units of hours, and the growth rates r_i are thus expressed in units of h^{-1}. The fits were performed using MATLAB 2016a, with its Non-linear Least Squares fitting method (code provided, see Data Availability statement). Typically, these parameters explain a very large majority of the variation in OD (Table B), except where there is effectively no growth (e.g. of S, R and N strains in high concentrations of the antibiotic combination).

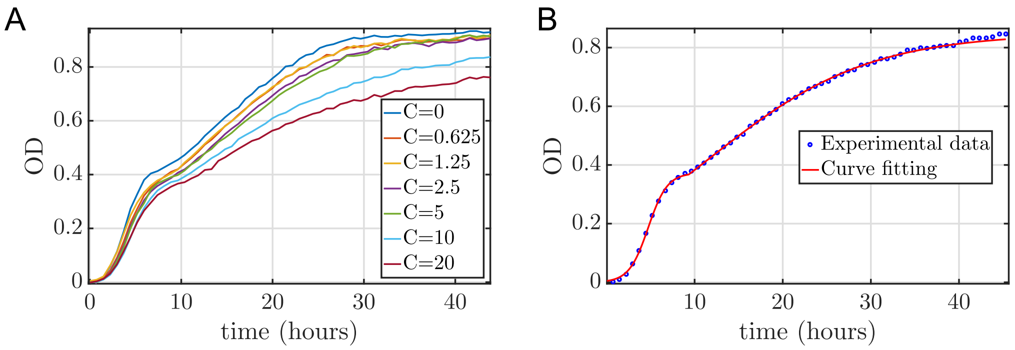

Fig B. Example growth curves used for parameter estimation. A) Example growth curves of a rifampicin-resistant clone under rifampicin treatment, for several drug concentrations, C (mg/l). B) Curve fitting for $C = 10$ mg/l of the rifampicin treatment for the rifampicin-resistant strain, resulting in estimates $k_R^{(1)} = 0.372689$ [95% CI: (0.3652, 0.3801)], $r_R^{(1)} = 0.921300$ [95% CI: (0.8678, 0.9748)], $k_R^{(2)} = 0.848618$ [95% CI: (0.8038, 0.8935)], $r_R^{(2)} = 0.110723$ [95% CI: (0.0998, 0.1216)], $R_R^2 = 0.99966$. Parameter estimates and R^2 for the other curves are shown in Fig D and Table B, respectively. OD–optical density.

Table B. R^2 values obtained from fitting Eq. (S15) to the growth curve data in Fig A. Model fitting was performed using the approach illustrated in Fig B for the strains S, R, N, and D.

Treatment	Transfer	Concentration (mg/l)	R_R^2	R_N^2	R_D^2	R_S^2
no antibiotic	1–6	0.31250	0.99957	0.99925	0.99907	0.99957
rifampicin	1	0.625	0.99960	0.99738	0.99966	0.99956
rifampicin	2	1.25	0.99964	0.99625	0.99958	0.99244
rifampicin	3	2.50	0.99958	0.98607	0.99946	0.99552
rifampicin	4	5	0.99956	0.99822	0.99967	0.99947
rifampicin	5	10	0.99966	0.99725	0.99969	0.94550
rifampicin	6	20	0.99933	0.99165	0.99974	0.37149
nalidixic acid	1	0.625	0.99959	0.99955	0.99953	0.99945
nalidixic acid	2	1.25	0.99974	0.99953	0.99964	0.99948
nalidixic acid	3	2.50	0.99763	0.99957	0.99944	0.99309
nalidixic acid	4	5	0.98397	0.99958	0.99970	0.98801
nalidixic acid	5	10	0.97461	0.99949	0.99970	0.96445
nalidixic acid	6	20	0.96781	0.99970	0.99932	0.95763
combination	1	0.625	0.99948	0.99940	0.99950	0.99913
combination	2	1.25	0.99962	0.99939	0.99964	0.99924
combination	3	2.50	0.97307	0.99931	0.99963	0.95726
combination	4	5	0.97735	0.98672	0.99960	0.88433
combination	5	10	0.93615	0.98740	0.99955	0.19791
combination	6	20	0.97295	0.97101	0.99955	0.35897

In order to include the diauxic-like behaviour in the simulation model, we switch between the two growth regimes when the total population size $n_T(t)$ reaches a threshold n_T^c. This threshold is related to the n_i^c obtained from the fits of single-strain growth experiments to the diauxic-like behaviour in Eq. (S10) as we will explain next.

The time t_i^c and population size n_i^c at which the growth switches from $n_i^{(1)}(t)$ to $n_i^{(2)}(t)$ is different for every strain, treatment, and drug concentration considered. Note that these parameters are obtained from growth experiments with strains grown on their own, whereas multiple strains are present in the simulation model. We therefore use the n_i^c distributions to choose a single time point at which to switch regimes for the entire population based on the procedure outlined below. Fig C shows the distribution of n^c, the switching population values, obtained for the different treatments. Each graph shows a histogram of the values, n^c, obtained from the different drug concentrations and strains for the given treatment. Since there are 6 drug concentrations as each growth experiment proceeds, and 4 different strains, this results in 24 values for n^c per treatment. Evidently, for experiments in which no drug is given we only obtain four values of n^c (one for each strain). We do not show the corresponding histogram.

Each of the histograms is bimodal, with one peak close to $n^c = 0$, and the other at $n^c \approx 0.3$ on the optical-density scale. The left peak at $n^c \approx 0$ corresponds to strains incapable of growing in those environments (e.g. strain S for high values of antibiotic concentration, C). Taking the average across this distribution would therefore not be representative of growing

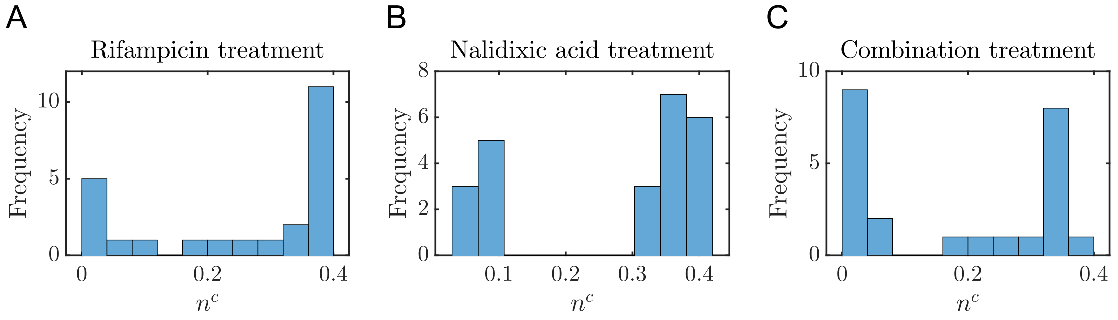

Fig C. Histograms of n^c obtained from fitting the two-stage 'diauxic-like' growth curves (Eqs. (S10) and (S11)) to experimental data; rifampicin, nalidixic acid, combination treatments in panels A, B and C, respectively. Each graph includes all the values (24 in total) of n_i^c obtained for each drug concentration and strain.

Table C. Switching value, n_T^c, for the different antibiotic treatments.

Treatment	Switching n_T^c
No antibiotic	0.4108
Rifampicin	0.3417
Nalidixic acid	0.3709
Combination	0.3154

strains. Instead, we estimate n_T^c as the mean value obtained from the peak on the right in each histogram, i.e. cases where strains were capable of growing. Results are shown in Table C. The value obtained in this way differs between the different treatments. For the case of no antibiotic treatment we use the average over the four strains as threshold value n_T^c.

Parameters estimated in this way are given in Fig D. As these parameters have been estimated from OD growth curves, they have so far been expressed in units of OD. In the next section, we will describe the relationship between OD and the number of bacteria.

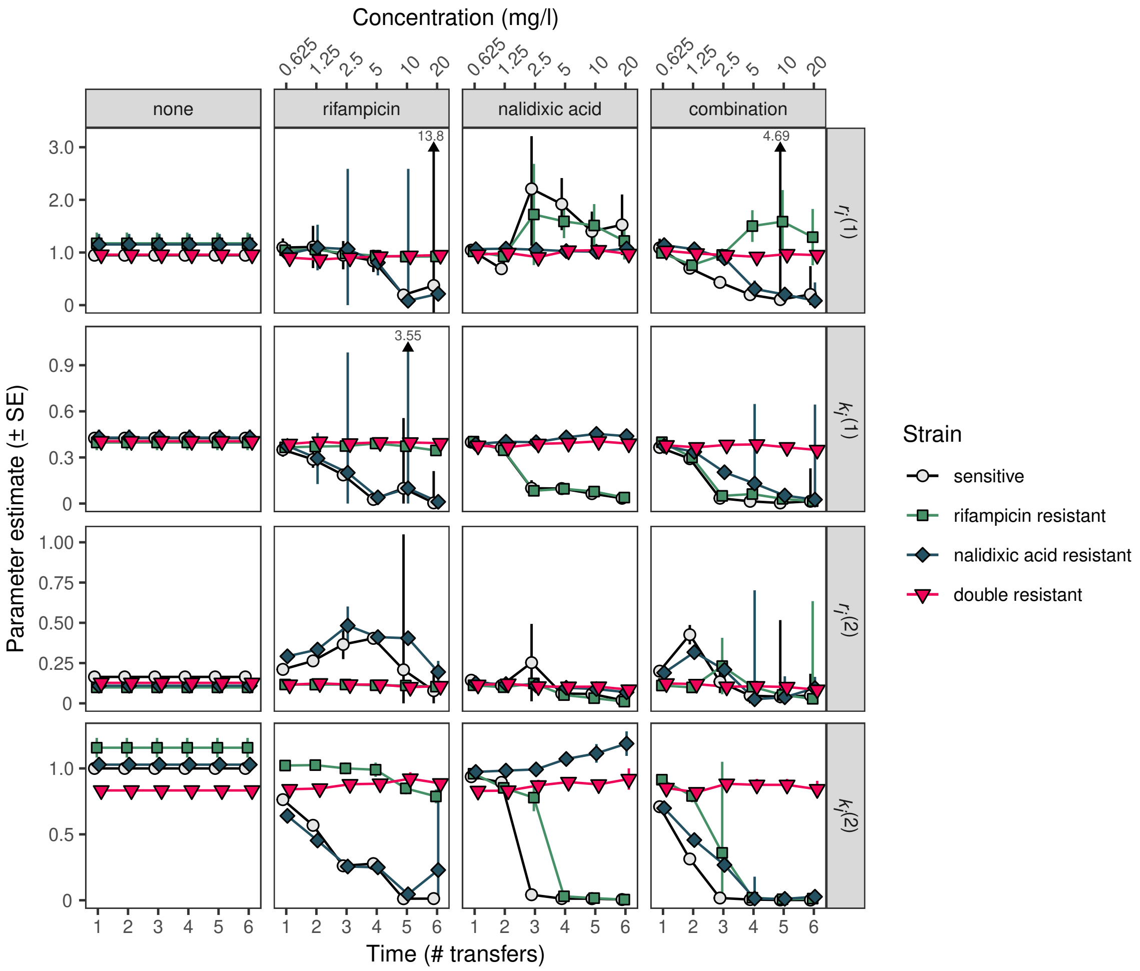

Fig D. Parameter estimates for $k_i^{(j)}$ and $r_i^{(j)}$ from growth curve fitting used in simulations (See Figs A and B). Error bars represent the standard error of the estimate across five independent strains (upper bounds of extreme error bars have been truncated as indicated by an arrow and its value; lower bounds are truncated at zero). Points have been displaced on the horizontal axis for clarity.

Relationship between optical density and bacterial population size

To simulate population dynamics, the parameters we have estimated need to be expressed in terms of numbers of bacteria, rather than in units of OD. We therefore determined the relationship between OD and the number of bacteria present (Fig E). Following growth in liquid MH (with and without antibiotics, as in the selection experiment), serial dilutions were plated on 90 mm Petri dishes containing LB agar (without antibiotics), and colony forming units (CFUs) were counted following overnight growth. We compared the fit of a linear ($y = mx + b$) and non-linear ($y = mx^a + b$) relationship between $\log_{10}$(CFUs) and $\log_{10}$(blanked OD). Both models were fit using Bayesian non-linear regression using `brm()` from the `brms` package [12, 13] in R [14]. Parameter estimates for both models are shown below in Table D. Model comparison was performed using the expected log pointwise predictive density calculated (ELPD) using leave-one-out (PSIS-LOO) cross-validation with `loo_compare()` [15]. The linear and non-linear models had equivalent predictive power when corrected for the number of parameters, therefore the simpler linear model was used to characterise the data (plotted in Fig E). Computing a Bayesian R^2 [16] using the `bayes_R2()` function suggests that the linear model provides a good fit to the data [Bayes $R^2 = 0.798$, 95% CI $(0.790, 0.804)$].

We also performed kill curves to quantify death that occurs when populations first experience antibiotic treatment. This was performed by pin replicating an overnight culture (1/200 dilution) of the sensitive ancestor into antibiotic containing medium (0.625, 1.25, 2.5, 5, 10, 20 mg/l of each antibiotic treatment). Population sizes were estimated by plating dilutions on LB agar after 2, 4, 6, 8, and 24 h of exposure (Fig E). In absolute terms, death due to antibiotic exposure was negligible for the rifampicin and combination treatments, but occurred for the concentrations achieved on day three and later for the nalidixic acid treatment. In relative terms, the number of bacterial cells killed by antibiotics is small compared to the bottlenecks imposed every seven generations, which reduce population size to 1/200.

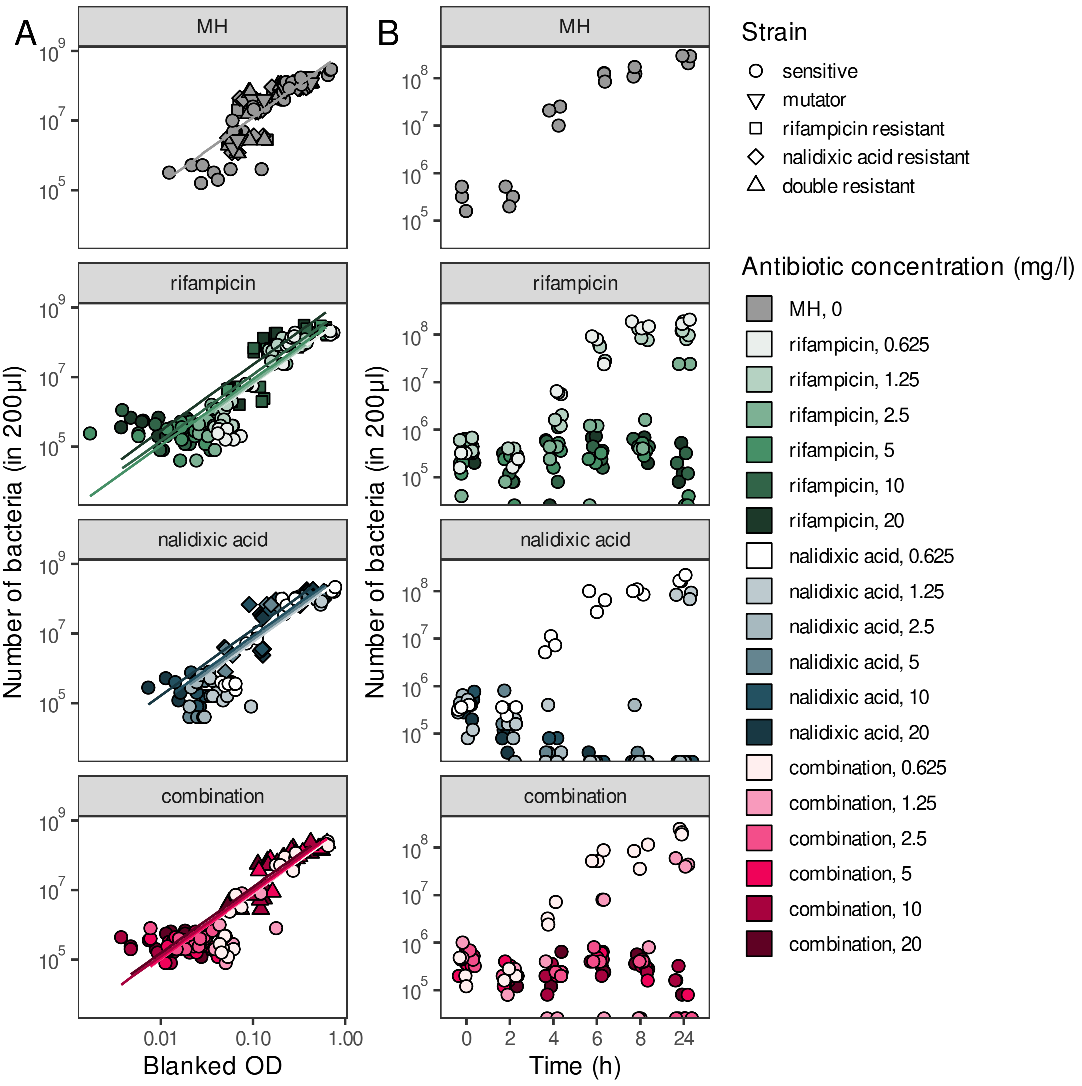

Fig E. Relationship between optical density and bacterial colony forming units. A) Blank corrected optical density (Blanked OD) and number of bacteria (measured by colony forming units) in antibiotic concentrations experienced during the selection experiment. Lines indicate the linear regression model fit to the data shown in the first model of Table D. B) Time series of sensitive bacterial populations exposed to antibiotic concentrations experienced during the selection experiment.

Table D. Bayesian non-linear regression parameters describing two possible log-log relationships between blank corrected optical density (mOD = OD rescaled by a factor of 1000) and colony forming units (CFU) using data from Fig E. Models were fit using `brm()` from the brms package. Both models had equivalent predictive power as measured by ELPD using the LOO cross-validation method.

Regression model of the form $\log_{10}(\text{CFU}) = a \log_{10}(\text{mOD}) + b_{\text{none}} + b_i$

	Estimate	Standard error
ELPD_{LOO}	-125.0	14.5
p_{LOO}	8.4	1.2
LOOIC	250.0	29.1

Parameter	Estimate	Estimate error	95% CI
a	1.895	0.065	$(1.772, 2.026)$
b_{none}	3.297	0.147	$(3.005, 3.581)$
$b_{\text{rifampicin}}$	-0.302	0.099	$(-0.499, -0.109)$
$b_{\text{nalidixic acid}}$	-0.345	0.112	$(-0.565, -0.123)$
$b_{\text{combination}}$	-0.197	0.109	$(-0.415, 0.018)$
$b_{\text{none:concentration}}$	-0.010	0.988	$(-1.956, 1.926)$
$b_{\text{rifampicin:concentration}}$	0.286	0.080	$(0.132, 0.443)$
$b_{\text{nalidixic acid:concentration}}$	0.187	0.087	$(0.022, 0.364)$
$b_{\text{combination:concentration}}$	0.098	0.088	$(-0.072, 0.268)$

Regression model of the form $\log_{10}(\text{CFU}) = a \log_{10}(\text{mOD})^c + b_{\text{none}} + b_i$

	Estimate	Standard error
ELPD_{LOO}	-123.9	15.2
p_{LOO}	9.2	1.5
LOOIC	247.7	30.3

Parameter	Estimate	Estimate error	95% CI
a	2.990	0.537	$(1.990, 4.069)$
c	0.762	0.093	$(0.607, 0.968)$
b_{none}	2.081	0.600	$(0.906, 3.231)$
$b_{\text{rifampicin}}$	-0.281	0.100	$(-0.475, -0.086)$
$b_{\text{nalidixic acid}}$	-0.316	0.112	$(-0.532, -0.092)$
$b_{\text{combination}}$	-0.168	0.107	$(-0.385, 0.038)$
$b_{\text{none:concentration}}$	0.012	0.991	$(-1.918, 1.970)$
$b_{\text{rifampicin:concentration}}$	0.282	0.079	$(0.126, 0.432)$
$b_{\text{nalidixic acid:concentration}}$	0.169	0.087	$(0.001, 0.341)$
$b_{\text{combination:concentration}}$	0.084	0.084	$(-0.080, 0.253)$

Simulating the experiments

Simulation conditions are given in Table E. We use the values $k_i^{(1)}, r_i^{(1)}, k_i^{(2)}, r_i^{(2)}$ of each strain and treatment, and n_T^c obtained as defined above, for simulations of the stochastic model. Simulations are started from an initial population of 5.71×10^6 sensitive bacteria (type S); a frequency u of these are mutators and the remaining fraction $(1 - u)$ consists of wild-type bacteria. In the simulations we use $\mu_R = 6.7 \times 10^{-9}$ and $\mu_N = 7.4 \times 10^{-10}$, as motivated in the main text. Mutators have a 80-fold increased mutation rates, μ_R and μ_N, compared to the wildtype.

Table E. Simulation conditions for resistance evolution simulations.

Parameter	Value
Replicates	1000
Initial population size	5.71×10^6
Mean proportion transferred during dilution step	0.005
Duration between dilution steps	22 h
Length of time per time-step (Δt)	0.01 h
Mutation rate to rifampicin resistance (μ_R)	6.7×10^{-9} mutations/replication
Mutation rate to nalidixic acid resistance (μ_N)	7.4×10^{-10} mutations/replication
Mutator effect	80-fold increase

The simulated experiment consists of six days of growth, where the concentration of antibiotic(s) is initially 0.625 on day 1, and doubled on each of the days 2 to 6. This procedure was chosen to reflect the experimental conditions. The length of each time-step of the simulation, Δt, is expressed in units of hours (h). It has to be sufficiently small so that the simulations maintain precision and capture the behaviour of the original continuous-time model. We have set $\Delta t = 0.01$ h, which is much smaller than the characteristic time of *E. coli* division, which is approximately 0.3–0.6 h [17]. this choice approximates simulations of the full model in continuous time to a good accuracy, while leading to a considerable reduction of the simulation time. In Fig F, we compare the distributions of the number of individuals of each strain obtained from these discrete-time simulations with simulations of the original continuous-time model. The discrete-time simulations approximate the original model with good accuracy. For the simulation conditions used in this figure, the simulation time of one single run (i.e. 22 h of the experiment) is approximately 500 times faster with our approach relative to the Gillespie algorithm on the same computing hardware.

An important aspect to take into account is the dilution carried out in the laboratory experiments at the end of each day, when $1/200$ of the population is carried forward to the next day, and the rest discarded. In the evolution literature, this is referred to as a population bottleneck. In simulations we cycle through all members of the population at the end of each (simulated) day and retain each individual with probability $1/200$; otherwise the individual is removed. This represents an independent Bernoulli event on the level of individual bacteria.

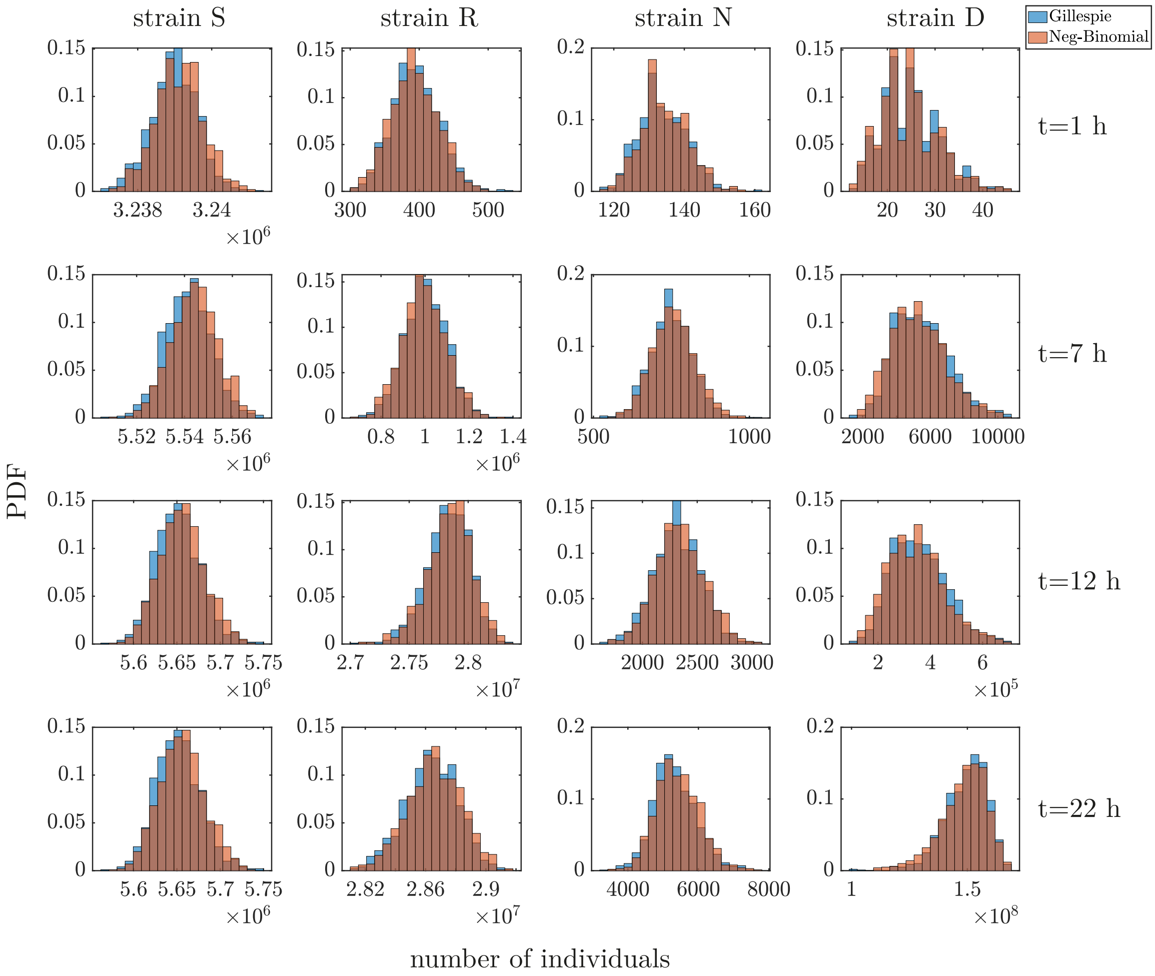

Fig F. Distributions of the number of individuals of each strain at different times obtained from continuous-time simulations (Gillespie algorithm) and discrete-time simulations (based on the negative binomial distribution). Data from simulations of the continuous-time model are shown as blue bars, those from the discrete-time approach as light orange bars. Dark brown colour indicates an overlap of both types of bars. The simulation parameters used are the parameter estimates from day 4 of the combination treatment (see Fig D). We set an initial number of wild-type individuals equal to $n_S = 2.8 \times 10^6, n_R = 100, n_N = 100$, and $n_D = 10$. For the discrete-time approach we set $\Delta t = 0.01$. The distributions were constructed over 1000 runs.

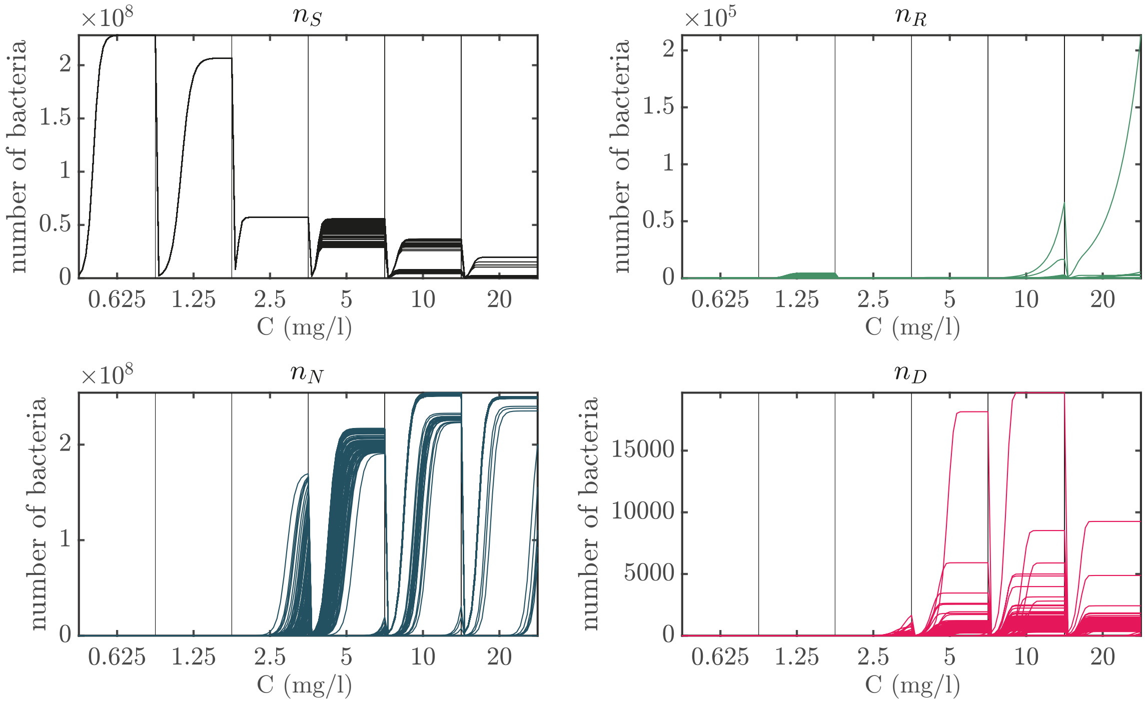

Fig G. Simulated growth curves for nalidixic acid treatment. Each panel shows 100 independent simulation runs. The data shown aggregates wildtype and mutator bacteria. C–antibiotic concentration (mg/l). Initial condition: 5.71×10^6 sensitive bacteria, with a frequency $u = 0.3$ of mutators. Curves for each value of C were simulated over 22 h. We have used $\Delta t = 0.01$ h.

The number of each type i carried forward follows a binomial distribution with mean $\mu = n_i/200$ and variance $\sigma^2 = n_i \times 1/200 \times 199/200 = n_i \times 4.975 \times 10^{-3}$, where n_i is the number of type i individuals. This allows the possibility that a strain type goes extinct if it arises too late into the growth cycle to achieve appreciable frequency. An example of the growth curves obtained from the simulations, including the dilution step, is presented in Fig G.

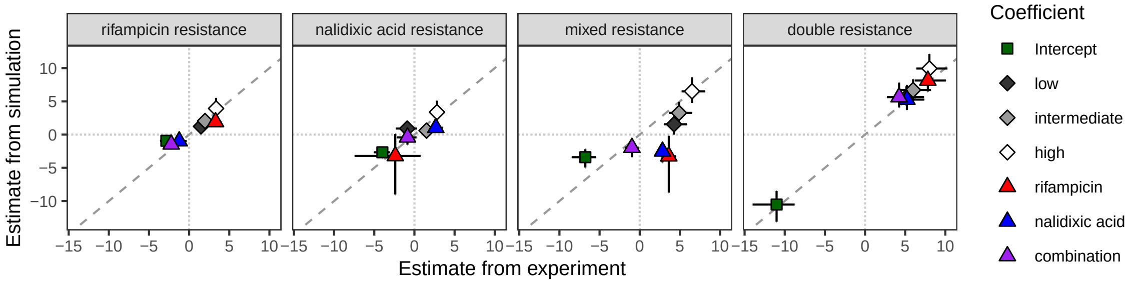

Fig H. Comparison of coefficients from Bayesian categorical models fitted to simulation and experimental data. Dashed diagonal line indicates 1:1 line; points falling on this line indicate coefficients are equal in both simulation and experiment.

Comparison between simulation and experimental results

To determine concordance between simulations and experimental results, we fit a Bayesian categorical model to the simulation results and compared the estimated coefficients. The same statistical model and priors were used to analyse the simulation data as were used in the analysis of the experimental results, with the exception of an absence of random effects of 'position' (see S1 Appendix). As for the experimental results, the full model with the interaction did not provide a better fit than the main effects model (WAIC 1486.5 ± 52.5 SE vs. 1515.0 ± 53.1 SE). The estimated coefficient obtained from fitting the main-effects model to the output from the simulations are therefore given in Table F. Estimated coefficients from the simulation closely matched those from the experiment (Fig H), with the exception of the 'mixed resistance' category, which the tended to estimate a different sign for the effects of the nalidixic acid and combination treatments.

Other simulation conditions

Simulating different antibiotic dose escalation regimes

In our experiments and main set of simulations, we have considered a particular dose escalation regime. Here we use the simulation model to predict whether other dose escalation regimes would affect the role of mutators in multi-resistance evolution. For example, if the concentration of the combination treatment increases faster, the sensitive and single-resistant strains will have less opportunity to acquire sequential resistance mutations (or conversely, if the concentration rises more slowly, there will be more opportunity for multi-resistance). To simulate reaching MIC at an earlier time, we time-shifted the parameter values relative to the original simulations (e.g. for MIC occurring on the third transfer instead of the fifth, the new values for transfers 1–4 corresponded to the parameters estimated for the 3rd, 4th, 5th (MIC), and 6th transfers of the original simulations). For simulations with MIC occurring later than the original

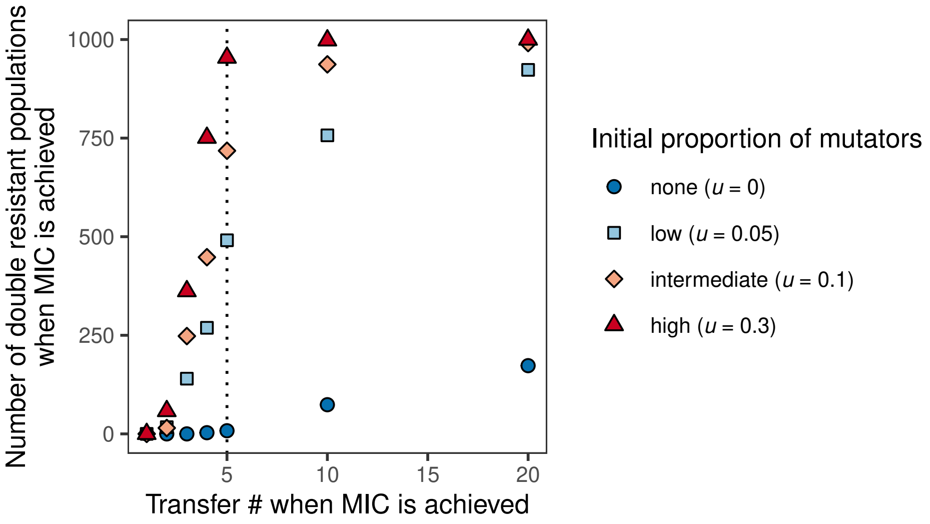

Fig I. Simulations of combination treatment where MIC is achieved after different numbers of population transfers ($n = 1000$ replicate simulations). Dotted vertical line corresponds to the simulation conditions used in the main text (Figs 3 and 4)

simulations, the parameter values were 'stretched' (e.g. for MIC occurring on the tenth transfer instead of the fifth, the values used for transfers 1–10 corresponded to the original parameters estimated for the 1st, 1st, 2nd, 2nd, 3rd, 3rd, 4th, 4th, 5th, 5th, 6th and 6th transfers).

The overall proportion of multi-resistant populations increases with the time taken to reach MIC (Fig I). When MIC was achieved earlier, there is overall less multi-resistance, but it nevertheless occurs for the highest mutator frequency, even when achieved just before the first transfer. When MIC was achieved later, the model predicts that a proportion of purely wild-type populations should achieve multi-resistance when the duration of the experiment is doubled or quadrupled (6.4% or 18.9% of populations, respectively), but that a small proportion of mutators still results in vastly higher multi-resistance (76.3% and 92.2% of populations, respectively). While multi-resistance is reduced when MIC occurs sooner, populations with mutators can still evolve multi-resistance, even for very fast dose escalation.

Table F. Effects of initial mutator frequency and antibiotic treatment on resistance state observed at the end of the simulations. Coefficients come from fitting a Bayesian categorical regression model to the simulations. Treatment contrasts on the logit scale are shown. (* denotes 95% credible intervals excluding zero).

Resistance state	Coefficient	Estimate	95% credible interval	
rifampicin resistance	intercept	-1.08	(-1.58, -0.60)	*
	low	1.41	(0.87, 1.96)	*
	intermediate	2.09	(1.50, 2.70)	*
	high	3.66	(2.57, 4.97)	*
	rifampicin	2.03	(1.34, 2.71)	*
	nalidixic acid	-0.97	(-1.56, -0.40)	*
	combination	-1.34	(-1.99, -0.71)	*
nalidixic acid resistance	intercept	-2.89	(-3.80, -2.06)	*
	low	0.81	(-0.04, 1.62)	
	intermediate	0.90	(-0.10, 1.85)	
	high	2.45	(0.84, 4.00)	*
	rifampicin	-2.94	(-8.42, 0.38)	
	nalidixic acid	1.29	(0.45, 2.19)	*
	combination	-0.15	(-1.31, 0.91)	
mixed resistance	intercept	-3.37	(-4.89, -2.17)	*
	low	1.56	(-0.02, 3.30)	
	intermediate	3.30	(1.95, 4.89)	*
	high	6.14	(4.55, 8.07)	*
	rifampicin	-3.26	(-8.59, -0.12)	*
	nalidixic acid	-2.58	(-4.21, -1.21)	*
	combination	-2.37	(-4.01, -1.04)	*
double resistance	intercept	-11.34	(-14.33, -9.15)	*
	low	6.43	(4.81, 8.75)	*
	intermediate	7.38	(5.75, 9.72)	*
	high	10.44	(8.50, 13.01)	*
	rifampicin	8.36	(6.66, 10.50)	*
	nalidixic acid	5.31	(3.72, 7.36)	*
	combination	5.72	(4.12, 7.81)	*

Simulating a cost of resistance and logistic growth

Our simulations used parameter values estimated from growth curve data from resistant strains, and a two-stage population growth model to match the shape of growth curves under our environmental conditions (rather than logistic growth, as is typically used to model microbial growth). The estimated parameters did not always demonstrate a 'cost of resistance' (Fig D), i.e. the phenomenon that resistance to an antibiotic tends to be associated with a fitness deficit in environments where that antibiotic is not present [18] (though resistance need not be costly [19]). This was particularly evident in the antibiotic-free environment where single-resistant strains had a higher growth rate ($r_i^{(1)}$) than the sensitive strain (Fig D). We therefore tested whether we would observe similar patterns of resistance emergence if we imposed arbitrary growth parameters for single resistant types A and B and double resistant type D that reflect a cost of resistance and standard logistic growth (keeping other model parameters, e.g. mutation rate, the same). Growth parameters were chosen so that only strains that would be expected to have a benefit in a given environment actually do (i.e. type A has an advantage in environments with drug A, type B with drug B, Fig J). We reduced our two-stage model to a logistic growth model by setting $r_i^{(1)} = r_i^{(2)}$ and $k_i^{(1)} = k_i^{(2)}$.

Using these parameters, we again observe that mutators allow multi-resistance to arise in both single-drug and combination treatment environments (Fig J), as we observed in the original simulations (Fig 3 in the main text). Hence, the patterns of resistance emergence we observed are not specific to the parameter estimates obtained from our experiments. Moreover, within populations, the spread of double resistance follows the hitch-hiking of a mutator allele along with a single resistance mutation, which subsequently increases the mutation rate of the population sufficiently to observe the secondary resistance mutation (Fig J panels C–F). This matches the same trend as the original simulations (Fig 4A in the main text and S1 Fig).

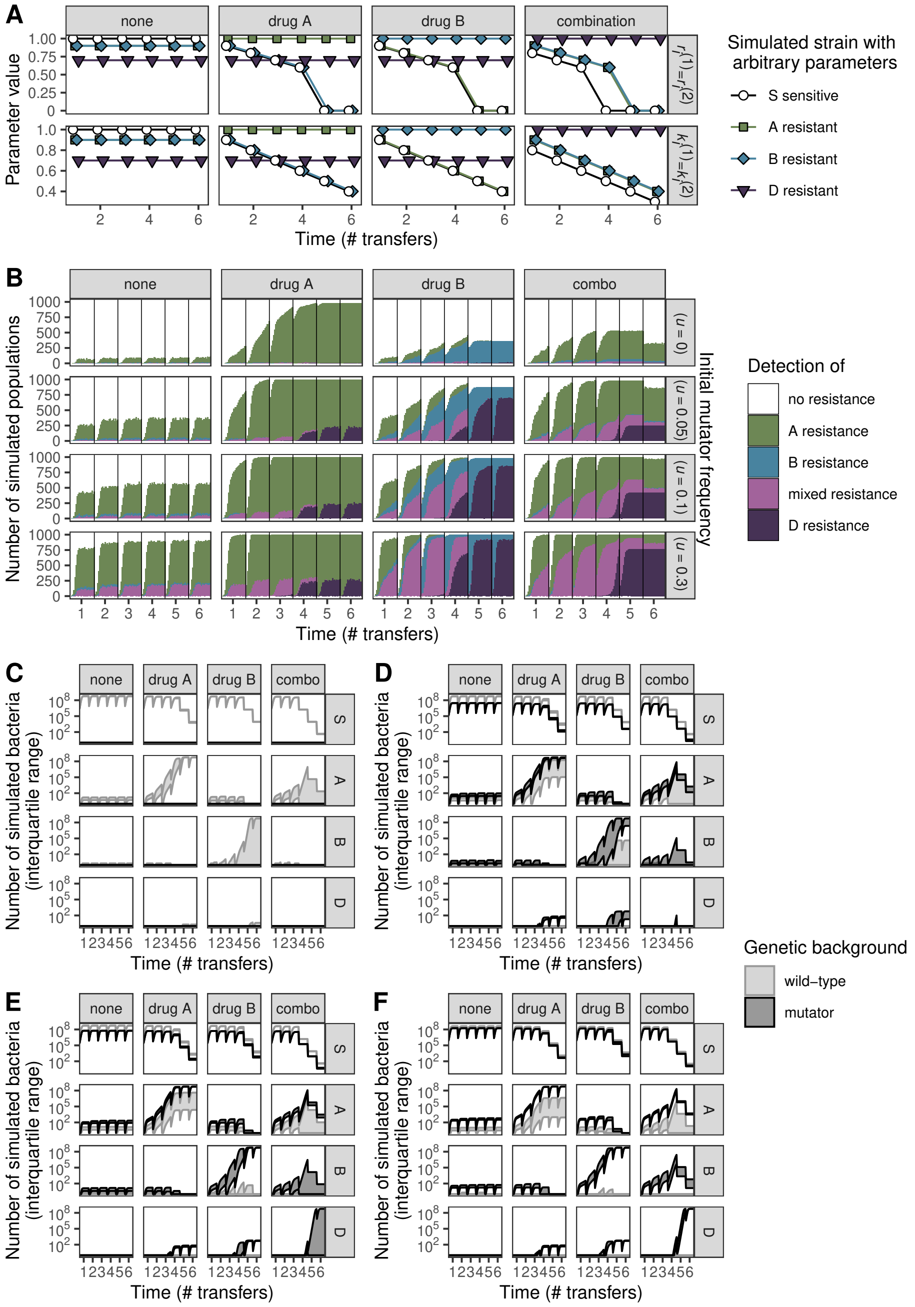

Fig J. Simulations with an imposed cost of resistance that also follow a logistic growth curve in pure culture (i.e. Eq. (S10), with $r_i^{(1)} = r_i^{(2)}$ and $k_i^{(1)} = k_i^{(2)}$) for $n = 1000$ replicate simulations. A) Parameter values were chosen so that simulated strains always exhibit a cost of resistance (points displaced on the horizontal axis for clarity). B) The proportion of populations with each strain type. C–F) Population dynamics of simulated resistance evolution for mutator frequencies 'none' (C, $u = 0$), 'low' (D, $u = 0.05$), 'intermediate' (E, $u = 0.1$), 'high' (F, $u = 0.3$).

Simulating synergistic and antagonistic antibiotic pairs

Much emphasis has been placed on characterising ‘synergistic’ or ‘antagonistic’ effects between pairs of antibiotics when used together [20–23]. Relative to additive combinations, where the amount of suppression imposed by each antibiotic is independent of the other drug(s) involved, synergistic combinations defined as those that are more suppressive than additive, and antagonistic combinations are less suppressive than additive. Classic results by Michel et al. [24] and Hegreness et al. [25] showed that synergistic interactions, although better at initially suppressing bacterial growth, were overall less robust against resistance evolution due to larger fitness benefits of single resistance mutations. In contrast, antagonistic interactions were more robust against resistance evolution as becoming resistant to one component drug reveals the full suppressive effects of the subsequent drug(s).

We therefore simulated antagonistic and synergistic antibiotic pairs with parameter values chosen to reflect these dynamics. Here we consider only the combination treatment, as synergy/antagonism only applies to this treatment. As in the previous section, we simulate logistic growth by setting $r_i^{(1)} = r_i^{(2)}$ and $k_i^{(1)} = k_i^{(2)}$; for convenience, we will drop the superscript notation here. Further, we treat types A and B as equivalent in fitness in the combination antibiotic environment. We define the degree of interaction between pairs as g, with $g = 0$ for additive pairs, $g < 0$ for synergistic pairs and $g > 0$ for antagonistic pairs. The the effect of the interaction on fitness is defined as the coefficient $\gamma = 10^g/(10^g + 1)$, $0 < \gamma < 1$.

Growth parameters of the single-resistant types, A and B, are then defined relative to the double-resistant type D, i.e. $r_{A,B} = r_D - (r_D - r_S)\gamma$ and $k_{A,B} = k_D - (k_D - k_S)\gamma$. Values of r_i and k_i are given in Fig K panel A; all other parameter values are as given in Table E. For the additive case, $g = 0$ and $\gamma = 1/2$, thus the growth parameters of the single-resistant types are half way between the sensitive and double resistant types. For synergy, the relative growth parameters of the single resitant types are greater than the additive case, and for antagonism, less than the additive case.

As before, we simulate populations where mutators are initially present. Consistent with the observations of others [24, 25], our simulations indicate that synergistic combinations are less robust against multi-resistance evolution, and antagonistic combinations are more robust (Fig K). The presence of mutators facilitates multi-resistance evolution across all synergistic combinations, and an increase in the frequency of mutators reduces the robustness of slightly antagonistic combinations. In addition, for synergistic combinations, we observe some degree of multi-resistance evolution in wild-type only populations, although the average number of multi-resistant individuals is roughly an order of magnitude lower than when mutators are present.

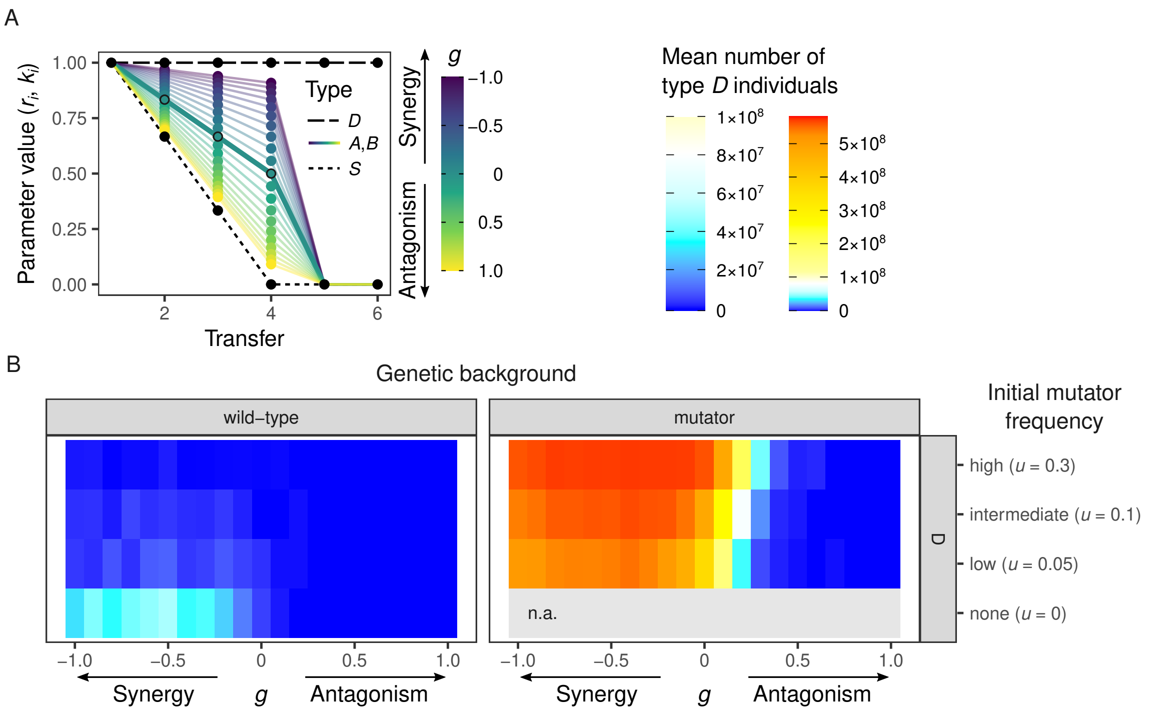

Fig K. Simulating synergistic and antagonistic antibiotic pairs affects the production of type D double resistant individuals arising in the combination environment. A) Growth parameters for additive ($g = 0$, highlighted points), synergistic ($g < 0$), and angatonistic ($g > 0$) antibiotic pairs. As in Fig J, strains follow a logistic growth curve in pure culture (i.e. Eq. S10, with $r_i^{(1)} = r_i^{(2)}$ and $k_i^{(1)} = k_i^{(2)}$). B) Average number of type D individuals after six transfers for $n = 1000$ replicate simulations.

Simulating very large population sizes

Our simulations have shown that mutators contribute to multi-resistance evolution because they increase the supply of resistance mutations, allowing independent resistance mutations to be acquired sequentially. However, mutational supply also depends on population size, which raises the possibility that multi-resistance may also evolve in large populations without mutators. We used our simulation model to determine how large a purely wild-type population would need to be to observe multi-resistance without mutators. Fig L shows the average proportion of each type of strain under different maximum population sizes after six days of simulated evolution. Multi-resistance reached appreciable frequencies for maximum population sizes between 5.71×10^{10} (combination treatment) and 5.71×10^{13} (no antibiotic treatment), which in all cases is orders of magnitude larger than the population size of 5.71×10^{8} in our experimental conditions. Bacterial population sizes in infections are typically smaller than this range [26, 27], although not always [28, 29]. Commensal populations, however, can achieve population sizes in the range of 10^{10} or more [30, 31], and 10^{13} bacteria is comparable in size to the entire human-associated microbiome [32]. Thus, multi-resistance may also be found without mutators in the largest microbial populations [although these populations also often contain mutators, 33].

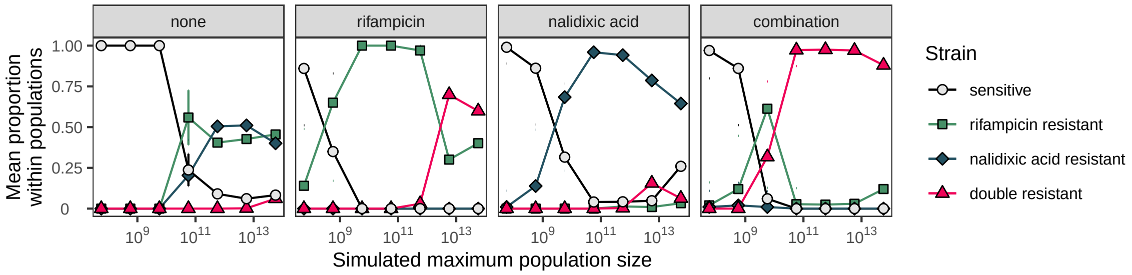

Fig L. Simulations of multi-resistance in the absence of mutators indicates multi-resistance can also evolve in very large populations. Proportion of each strain type (±SD, $n = 1000$ replicate simulations) found within simulated populations.

References

[1] Zheng Q. Progress of a half century in the study of the Luria–Delbrück distribution. Mathematical Biosciences. 1999 Nov;162(1):1-32.

[2] Bozic I, Nowak MA. Timing and heterogeneity of mutations associated with drug resistance in metastatic cancers. Proceedings of the National Academy of Sciences. 2014 Nov;111(45):15964-8.

[3] Durrett R. Branching Process Models of Cancer. Cham, Switzerland: Springer International Publishing; 2015.

[4] Bokes P, Singh A. A modified fluctuation test for elucidating drug resistance in microbial and cancer cells. European Journal of Control. 2021 Nov;62:130-5.

[5] Berríos-Caro E, Gifford DR, Galla T. Competition delays multi-drug resistance evolution during combination therapy. Journal of Theoretical Biology. 2021 Jan;509:110524.

[6] Gillespie DT. A general method for numerically simulating the stochastic time evolution of coupled chemical reactions. Journal of Computational Physics. 1976 Dec;22(4):403-34.

[7] Gillespie DT. Exact stochastic simulation of coupled chemical reactions. Journal of Physical Chemistry. 1977 Dec;81(25):2340-61.

[8] Gillespie DT. Approximate accelerated stochastic simulation of chemically reacting systems. The Journal of Chemical Physics. 2001;115(4):1716-33.

[9] Cavallero A, Eftimiadi C, Radin L, Schito GC. Suppression of tricarboxylic acid cycle in *Escherichia coli* exposed to sub-MICs of aminoglycosides. Antimicrobial Agents and Chemotherapy. 1990 Feb;34(2):295-301.

[10] Wang CH, Koch AL. Constancy of growth on simple and complex media. Journal of Bacteriology. 1978 Dec;136(3):969.

[11] Sezonov G, Joseleau-Petit D, D'Ari R. *Escherichia coli* Physiology in Luria-Bertani Broth. Journal of Bacteriology. 2007 Dec;189(23):8746-9.

[12] Bürkner PC. brms: An R Package for Bayesian Multilevel Models Using Stan. Journal of Statistical Software. 2017;80(1):1-28.

[13] Bürkner PC. Advanced Bayesian Multilevel Modeling with the R Package brms. The R Journal. 2018;10(1):395-411.

[14] R Core Team. R: A Language and Environment for Statistical Computing. Vienna, Austria; 2019. Available from: `https://www.R-project.org/`.

[15] Vehtari A, Gelman A, Gabry J. Practical Bayesian model evaluation using leave-one-out cross-validation and WAIC. Statistics and Computing. 2017;27(5):1413-32.

[16] Gelman A, Goodrich B, Gabry J, Vehtari A. R-squared for Bayesian Regression Models. American Statistician. 2019;73(3):307-9.

[17] Helmstetter CE, Cooper S. DNA synthesis during the division cycle of rapidly growing *Escherichia coli* B/r. Journal of Molecular Biology. 1968;31(3):507-18.

[18] Andersson DI. The biological cost of mutational antibiotic resistance: any practical conclusions? Current Opinion in Microbiology. 2006;9(5):461–465.

[19] Lenormand T, Harmand N, Gallet R. Cost of resistance: an unreasonably expensive concept. Rethinking Ecology. 2018 Dec;3:51-70.

[20] Bollenbach T. Antimicrobial interactions: mechanisms and implications for drug discovery and resistance evolution. Current Opinion in Microbiology. 2015;27:1-9.

[21] Baym M, Stone LK, Kishony R. Multidrug evolutionary strategies to reverse antibiotic resistance. Science. 2016;351(6268):aad3292.

[22] Barbosa C, Beardmore R, Schulenburg H, Jansen G. Antibiotic combination efficacy (ACE) networks for a *Pseudomonas aeruginosa* model. PLOS Biology. 2018;16(4):e2004356.

[23] Gjini E, Wood KB. Price equation captures the role of drug interactions and collateral effects in the evolution of multidrug resistance. eLife. 2021 Jul:e64851.

[24] Michel JB, Yeh PJ, Chait R, Moellering RC, Kishony R. Drug interactions modulate the potential for evolution of resistance. Proceedings of the National Academy of Sciences. 2008 Sep;105(39):14918-23.

[25] Hegreness M, Shoresh N, Damian D, Hartl D, Kishony R. Accelerated evolution of resistance in multidrug environments. Proceedings of the National Academy of Sciences. 2008;105(37):13977-81.

[26] Orenstein R, Wong ES. Urinary tract infections in adults. American Family physician. 1999;59(5):1225.

[27] Opota O, Croxatto A, Prod'hom G, Greub G. Blood culture-based diagnosis of bacteraemia: state of the art. Clinical Microbiology and Infection. 2015;21(4):313-22.

[28] Bingen E, Lambert-Zechovsky N, Mariani-Kurkdjian P, Doit C, Aujard Y, Fournerie F, et al. Bacterial counts in cerebrospinal fluid of children with meningitis. European Journal of Clinical Microbiology and Infectious Diseases. 1990;9(4):278-81.

[29] Stressmann FA, Rogers GB, Marsh P, Lilley AK, Daniels TWV, Carroll MP, et al. Does bacterial density in cystic fibrosis sputum increase prior to pulmonary exacerbation? Journal of Cystic Fibrosis. 2011 Sep;10(5):357-65.

[30] Savage DC. Microbial ecology of the gastrointestinal tract. Annual Review of Microbiology. 1977;31(1):107-33.

[31] Mason TG, Richardson G. *Escherichia coli* and the human gut: some ecological considerations. Journal of Applied Bacteriology. 1981 Aug;51(1):1-16.

[32] Sender R, Fuchs S, Milo R. Revised estimates for the number of human and bacteria cells in the body. PLOS Biology. 2016;14(8):e1002533.

[33] Ramiro RS, Durão P, Bank C, Gordo I. Low mutational load and high mutation rate variation in gut commensal bacteria. PLOS Biology. 2020 Mar;18(3):e3000617.
