# Supplementary figures and images for: Mutators can drive the evolution of multi-resistance to antibiotics
Source: PLoS Genet. 2023 Jun 13;19(6):e1010791. doi: 10.1371/journal.pgen.1010791 (PMC10292718; doi:10.1371/journal.pgen.1010791)

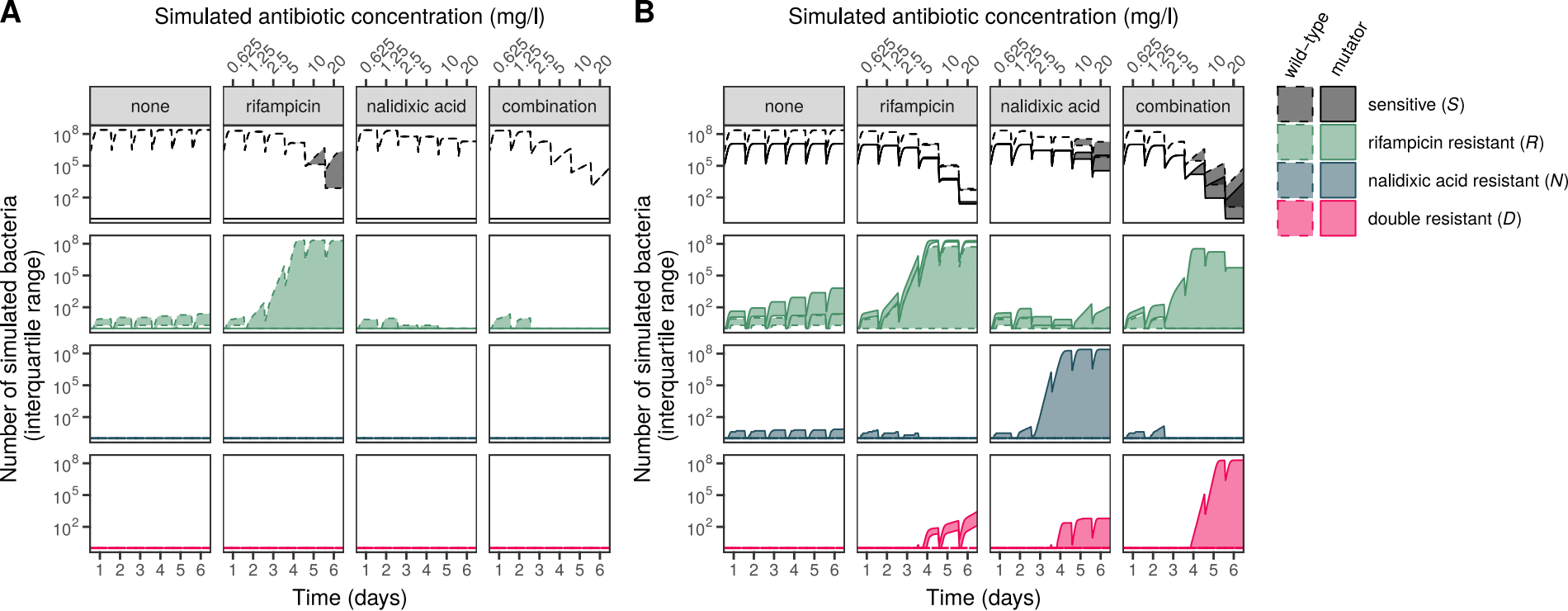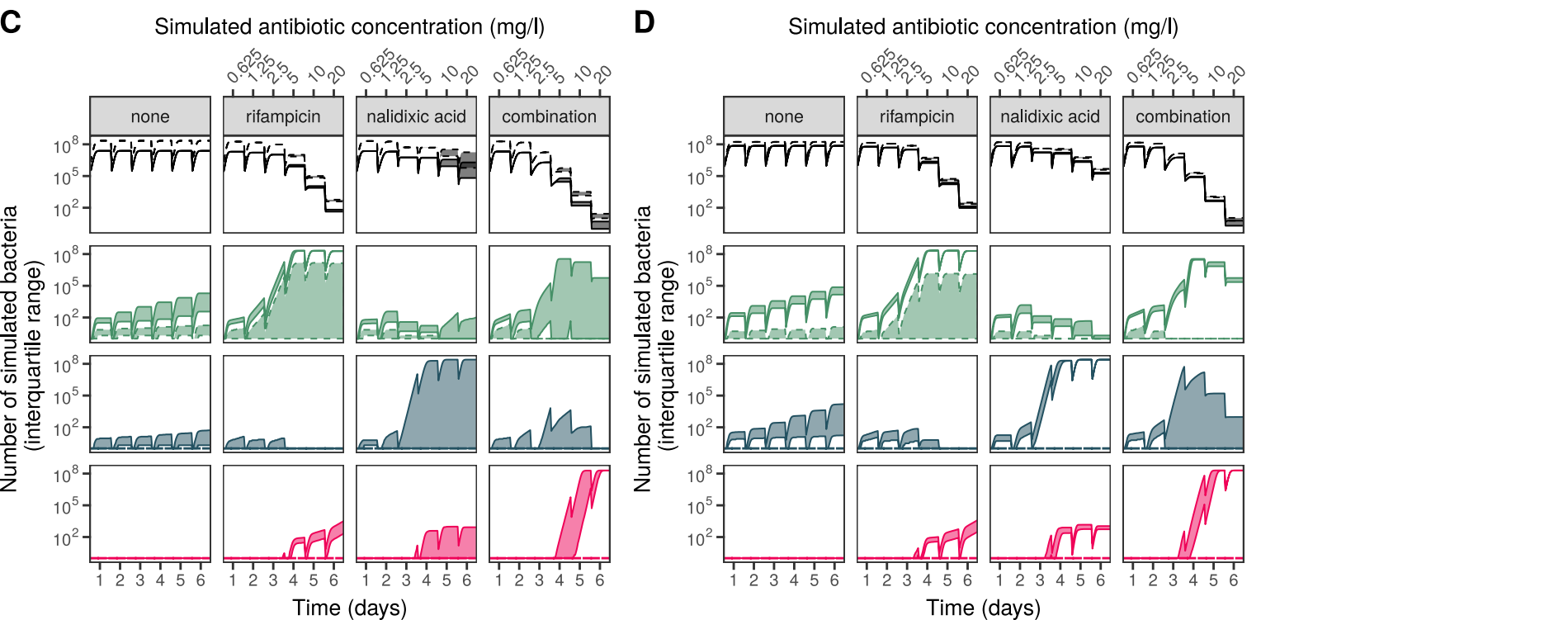

Supplement: S1 Fig — Interquartile range (IQR) of the number of bacteria of each resistance type over time for the four simulated treatments for n = 1000 replicate simulations. Areas indicate the interquartile range (25% and 75% quantiles) of the numbers of bacteria of each resistance type from n = 1000 replicate stochastic simulations. Panels A–D show different initial mutator frequencies: ‘none’ (u = 0), ‘low’ (u = 0.05), ‘intermediate’ (u = 0.1, as shown in main text Fig 4), ‘high’ (u = 0.3). (PDF) [file pgen.1010791.s006.pdf]

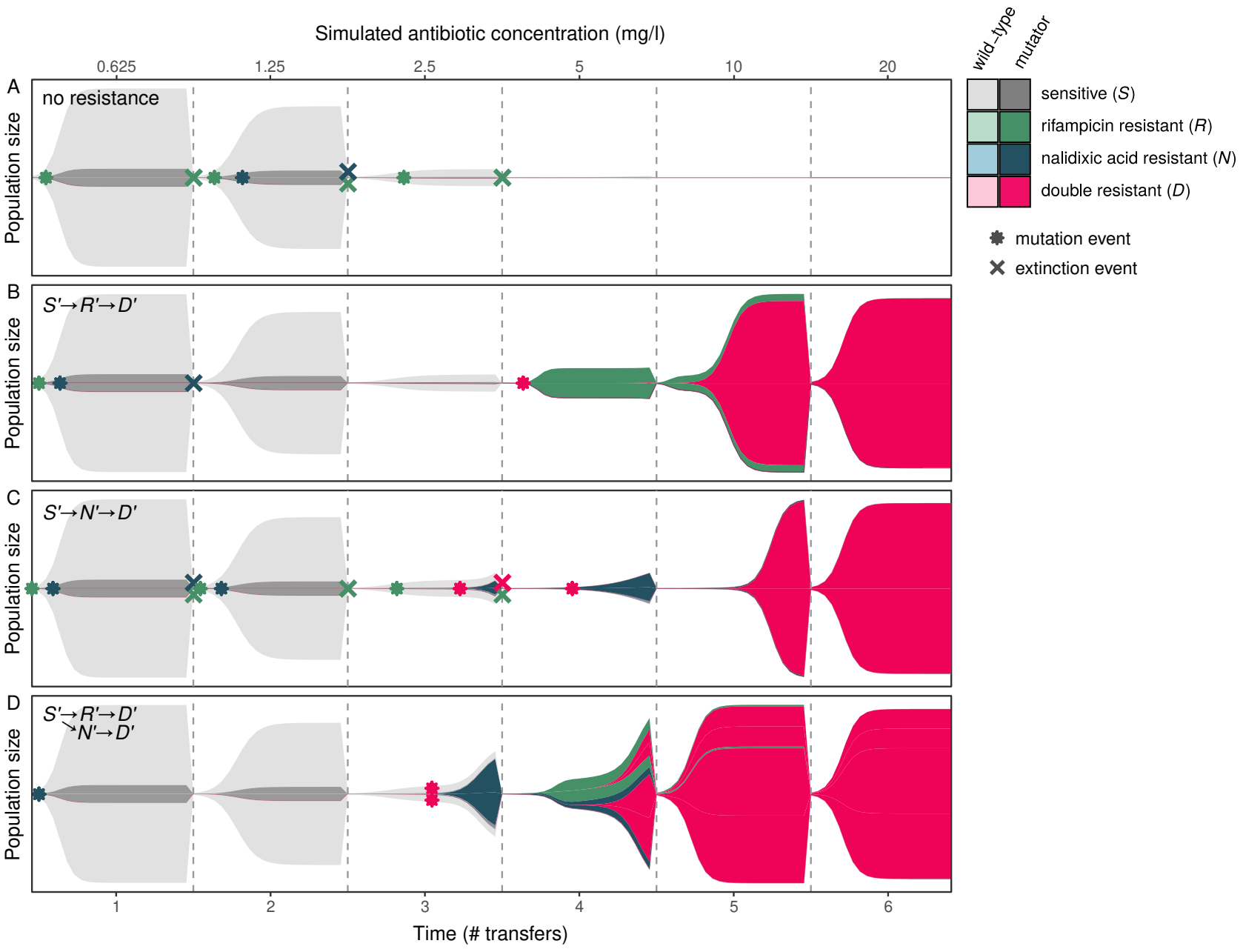

Supplement: S2 Fig — Dynamics are described as follows: A. Single resistance emerged, but failed to establish and ultimately no double resistance is observed. B. Rifampicin resistance establishes first, followed by double resistance (equivalent to main text Fig 4A). C. Nalidixic acid resistance establishes first, followed by double resistance. D. Rifampicin resistance and nalidixic acid resistance both established, followed by double resistance arising in both genetic backgrounds. Areas correspond to the population size of each type. Examples shown are individual replicates from the ‘intermediate’ initial mutator frequency (u = 0.1) treatment. (PDF) [file pgen.1010791.s007.pdf]
